# Occurrence of Pharmaceuticals and Endocrine Disrupting Compounds in Brazilian Water and the Risks They May Represent to Human Health

**DOI:** 10.3390/ijerph182211765

**Published:** 2021-11-09

**Authors:** Sérgio Francisco de Aquino, Emanuel Manfred Freire Brandt, Sue Ellen Costa Bottrel, Fernanda Bento Rosa Gomes, Silvana de Queiroz Silva

**Affiliations:** 1 Chemistry Department, Federal University of Ouro Preto (UFOP), Ouro Preto 35400-000, MG, Brazil; 2 Environmental and Sanitary Engineering Department, Federal University of Juiz de Fora (UFJF), Juiz de Fora 36036-900, MG, Brazil; emanuel.brandt@ufjf.edu.br (E.M.F.B.); sue.bottrel@ufjf.edu.br (S.E.C.B.); 3 Civil Engineering Graduate Programme, Federal University of Juiz de Fora (UFJF), Juiz de Fora 36036-900, MG, Brazil; fernanda.bento@engenharia.ufjf.br; 4 Biological Sciences Department, Federal University of Ouro Preto (UFOP), Ouro Preto 35400-000, MG, Brazil; silvana.silva@ufop.edu.br

**Keywords:** antibiotics, drinking water, contaminants of emerging concern, hormones, antimicrobial resistance, quantitative chemical risk assessment, resistance genes, resistant bacteria

## Abstract

The risks of pharmaceuticals and endocrine disrupting compounds (P&EDC) to the environment and human health are a current topic of interest. Hundreds of P&EDC may reach the environment, hence, there is a need to rank the level of concern of human exposure to these compounds. Thus, this work aimed at setting a priority list of P&EDC in Brazil, by studying their occurrence in raw and drinking water, calculating health guideline values (GV), and estimating the risks of population exposure to water intake. Data on the Brazilian pharmaceutical market as well as published data of the monitoring of Brazilian natural and drinking water have been collected by means of an exhaustive literature review. Furthermore, many foreign data were also collected to enable a comparison of the values found in Brazilian studies. A list of 55 P&EDC that have the potential to be found in Brazilian water is proposed, and for 41 of these a risk assessment was performed by estimating their margin of exposure (ME), by considering their occurrence in drinking water, and guideline values estimated from reported acceptable daily intake (ADI) data. For seven compounds the risk was deemed high (three estrogens and four anti-inflammatories), whereas for another seven compounds, it was regarded as an ‘alert’ situation. Although such risk analysis is conservative, since it has been calculated based on the highest reported P&EDC concentration in drinking water, it highlights the need to enhance their monitoring in Brazil to strengthen the database and support decision makers. An analysis of the occurrence of antimicrobial resistance agents (antibiotics, resistant bacteria, and resistance genes) in surface waters was also carried out and confirmed that such agents are present in water sources throughout Brazil, which deserves the attention of policy makers and health agents to prevent dissemination of antimicrobial resistance through water use.

## 1. Introduction

For nearly three decades, environmental monitoring of the so called organic “micropollutants” has received great attention due to clues of aquatic toxicity, genotoxicity, endocrine disturbance, induction of antimicrobial resistance, and other effects [1,2,3]. The term micropollutant is used because these compounds are typically found at trace concentrations, from ng/L to µg/L, in aquatic environments or other environmental matrices [2,4,5]. This group of contaminants includes pharmaceuticals of different classes (e.g., analgesics, antibiotics, lipid lowering agents, anti-inflammatories, and contraceptives), cleaning and personal care products (e.g., surfactants such as alkylphenol ethoxylates, fragrances such as tonalid, and antiseptics such as triclosan), as well as natural and/or synthetic hormones excreted by humans and other animals (e.g., estrone, estradiol, estriol, and ethinylestradiol) [4,6,7,8].

Currently, there is no consensus or sufficient evidence about the safety levels needed to prevent adverse effects of these compounds on the environment or human health. Thus, most of them are generally not included in environmental regulations or drinking water standards and, therefore, they are also called contaminants of emerging concern (CEC) [1,6,7,9,10]. A CEC must remain emerging as long as there is a lack of scientific information about its potential risk [9,10]. Importantly, for many CEC, what is emerging is the concern about their environmental effects and not the pollutant itself. Examples are alkylphenol ethoxylates (APEO), which have been used for over 70 years in various products, mainly cleaning products, and estrogen hormones (e.g., estradiol) which are naturally excreted by humans [5]. The emerging concern is, in part, due to advances in analytical methods, which includes improvements in analyte extraction and concentration techniques related to chromatography coupled with mass spectrometry. These improvements have enabled the detection of organic compounds in complex matrices and their quantification at very low concentrations, on the order of nanograms per liter (ng/L or 1 part per trillion, ppt) to picograms per liter (pg/L or 1 part per quadrillion, ppq) [2,5,11,12]. In other words, the thresholds that define the occurrence or absence of organic compounds in environmental samples has been gradually reduced, leading researchers to ask whether the presence of previously undetected contaminants in water represents a risk to human health.

The literature shows that raw and treated sewage discharge in water bodies represent an important route of water contamination by CEC. Such contaminants may be present in water derived from showers, washbasins, and laundries, as well as excreta from individuals which contain, particularly, oral medications and natural hormones. Direct disposal of medicinal drugs and other chemicals in sanitary facilities may also be an important contribution to sewage contamination with CEC [13,14,15]. Only a small fraction of CEC is removed in conventional sewage treatment plants (which often employ biological processes), so that water bodies are continuously contaminated with such compounds or their byproducts [2,3,4]. Another aspect that certainly contributes to the occurrence of CEC in water bodies, especially in Brazil, is the low coverage of sewage collection and treatment [12]. Hospital and industrial wastewater, disposal of solid waste, and agricultural land runoff also contribute to the occurrence of CEC in water bodies [1,3,14]. Some CEC, such as ethinylestradiol (pharmaceutical), bisphenol A (plasticizer), and nonylphenol (surfactant), may mimic the action of estrogen hormones and interfere with the biota endocrine system. These compounds are also called “endocrine disruptors” or “hormonally active agents” [16,17,18].

In Brazil, pioneering works on CEC in water bodies were published at the end of the 1990s by Ternes et al. [19] and Stumpf et al. [20], which monitored pharmaceuticals and endocrine disruptors in raw and treated sewage in a wastewater treatment plant and water sources in Rio de Janeiro. After these, other works reported monitoring data of several CEC, mainly P&EDC, in natural and drinking water (effluent of chlorination chambers at WTPs or tap water at consumer houses) at different seasons of the year, mainly in the states of Minas Gerais (MG) and São Paulo (SP) [21,22,23,24,25,26,27,28,29,30,31,32].

As far as we are aware, no country currently has regulations establishing limit concentrations in water intended for human consumption for pharmaceuticals, cleaning and personal care products, or natural estrogens. Australia is perhaps the only country that considers guideline values for some of these CECs in the context of water reuse for human supplies (Augmentation of Drinking Water Supplies) [33]. It should be noted that, in Australia, both the water potability standard (Australian Drinking Water Guidelines) [34] and the water reuse standard [33] are not mandatory. Such standards are presented in the form of guiding values that need to be evaluated in light of the diversity of regional or local factors and take into account economic, political, and cultural issues, including consumer expectations, and willingness and ability to pay. In this context, the Australian Water Reuse Guide [33] discusses the effects of endocrine disruptors, pharmaceuticals, cleaning products, and personal hygiene in more detail and presents guideline values based on human health risks, for several CEC, among which are 84 drugs and metabolites (28 antibiotics, 9 anti-inflammatory drugs, 7 beta blockers, 12 hormones, and 28 of other classes), 7 fragrances, and 42 chemical compounds from a miscellany that includes phthalic esters (or phthalates) and alkylphenols, which are recognized endocrine disruptors.

In the USA, in 2016, USEPA published the fourth list of contaminant candidates—CCL-4 (Contaminant Candidate List), listing chemical and microbiological contaminants that are currently not part of the drinking water standard in the country (National Primary Drinking Water Regulation) but which can occur in public water supply systems and, therefore, are candidates for future regulation [35]. In CCL-4, which contains 97 compounds or chemical groups, 13 compounds used in pharmaceuticals or cleaning and personal care products are listed. These are the estrogenic compounds 17-alpha-estradiol, 17-beta-estradiol, estriol, estrone, 17-alpha-ethinylestradiol, mestranol, equilenin, equilin, and norethindrone; the antibiotic erythromycin; the solvents N-methyl-2-pyrrolidone and 2-methoxyethanol; and nonylphenol surfactant [35]. USEPA is currently preparing the fifth contaminant candidate list (CCL-5), and the contributions received after public consultation are under evaluation. The CCL-5 draft keeps the estrogenic compounds 17-alpha-ethinylestradiol, 4-nonylphenol (all isomers) and bisphenol A already listed in CCL-4 [36].

Recently the European Parliament formally adopted the revised Drinking Water Directive, which entered into force on 12 January 2021. The Drinking Water Directive updated quality standards and introduced the watch list mechanism, including unprecedented P&EDC [37]. Bisphenol A (BPA) was added to the Directive with a maximum value limit of 2.5 µg/L [38], while β-Estradiol (E2) and nonylphenol (NP) have been included in the watch list, in order to respond to growing public concerns about the effects of endocrine disrupting compounds on human health through the use of drinking water.

Furthermore, one of the main concerns related to the occurrence of pharmaceuticals in natural water is the presence of antibiotics due to the increase in antimicrobial resistance they may cause. Indeed, there are many papers showing the spread of resistance genes (ARG) and resistant bacteria (ARB) in water contaminated with antibiotics [39,40,41]. Antibiotic molecules have been part of Earth’s microbiome long before the use of these substances for medical purposes [42]. Antibiotics can be naturally produced by soil microorganisms, not necessarily for bactericidal purpose but as intercellular signaling molecules [43] for keeping community homeostasis, regulating complex interactions, and metabolic pathways that take place in microbial consortia [44].

As discussed before, antibiotics and their metabolites can reach the water bodies through sewage systems, especially in those countries with low sanitation coverage where there is a lack of wastewater collection and treatment. In fact, polluted water may constitute a reservoir for disseminating antibiotic-resistance into the community. Despite the concerns related to the spread of antibiotic resistance (AR) directly from polluted water, this is not the only way for AR dissemination. Antimicrobial resistance genes (ARG) and antimicrobial resistant bacteria (ARB) can reach the environment from wild animal microbiomes and from the selective pressure of metals in the environment by a co-resistance phenomenon, which have been recently reported for superficial Brazilian water [45,46]. As a natural phenomenon, it can be inferred that antibiotic resistance spread in nature can be intrinsically controlled. However, the continuous input of extra antibiotics into the environment, has increased the quantity of ARB in water bodies, which may boost exchange of their genetic material to resistant and nonresistant bacteria, giving them the opportunity to express the new phenotypic ability.

Therefore, the spread of antibiotic resistance can be carried out not only by pathogenic bacterial but also commensal species living in the environment. The last can be a reservoir of ARG since these, as any other gene, can be acquired by pathogenic bacteria living close by, leading to health concerns. Fecal bacteria are also considered an important reservoir of ARG in aquatic environments and could horizontally transfer these genes to autochthonous bacteria when carried on transferable and/or mobile genetic elements such as integrons, plasmids, and transposons [47].

Specialists from the World Health Organization have developed a list of global priority pathogens, which contains the most important resistant bacteria at a global level for which there is an urgent need for new clinical treatments. Of critical concern, considered priority 1, are *Acinetobacter baumannii* and *Pseudomonas aeruginosa* (both carbapenem-resistant) and members of the Enterobacteriaceae family (carbapenem-resistant and 3rd generation cephalosporin-resistant). Priority 2 are *Enterococcus faecium* (vancomycin-resistant), *Staphylococcus aureus* (methicillin-resistant and vancomycin intermediate and resistant), *Helicobacter pylori* (clarithromycin-resistant), *Campylobacter* sp. and *Salmonella* spp. (fluoroquinolone-resistant), and *Neisseria gonorrhoeae* (3rd generation cephalosporin-resistant and fluoroquinolone-resistant). Finally, considered priority 3 are *Streptococcus pneumoniae* (penicillin-non-susceptible), *Haemophilus influenzae* (ampicillin-resistant), and *Shigella* spp. (fluoroquinolone-resistant) [48]. Therefore, investigation of genetic elements for antibiotic resistance and antibiotic resistant bacteria in water bodies is important to identify possible reservoirs of resistant microorganisms that could reach human and animal health throughout water consumption.

A previous survey carried out by Nascimento and Araújo [49], considering publications in the period between 1988 and 2013 on antimicrobial resistance in bacteria isolated from aquatic environments in Brazil, revealed that Gram-negative bacteria were the most studied microbial group (71.4%), and the *Aeromonas* spp. was the one most frequently (19.0%) found. The antimicrobials chloramphenicol (81.0%), gentamicin (76.2%), sulpha/trimethroprim (71.4%), ampicillin (61.9%), and tetracycline (71.4%) were the most investigated antibiotics; and the ones with higher prevalence of resistance were chloramphenicol (58.8%), sulpha/trimethroprim (78.5%), and ampicillin (84.6%). According to these authors, the reduced number of works in the North/Northeast region of Brazil is of particular concern, since in these areas it is common practice to store water to use in the dry season, which can lead to transmission of pathogenic and possibly multiresistant species [49]. More recently Reichert et al. [50], considering publications between 2000 and 2019, revealed that the antibiotics with higher bacterial resistance rates were tetracycline, amoxicillin, ampicillin, imipenem, gentamycin, and cephalothin, which are frequently employed to treat and prevent infections. According to them, the main sources of antibiotic resistance in Brazil are hospital, domestic, and agricultural wastes or wastewater.

Given the above and considering that hundreds of compounds may be classified as P&EDC, it is necessary to establish the real level of concern or relevance of such compounds in water in an attempt to prioritize them to help regulatory agencies and decision makers. This analysis requires studying their occurrence in water sources and drinking water (sampled at the WTP exit or at the consumer’s tap), as well as assessing potential risks associated with the exposure to these compounds through water consumption [51]. In this sense, this work aims at setting a priority list of P&EDC in Brazil by means of a Quantitative Chemical Risk Assessment (QCRA), from an initial list of compounds that was produced considering both sales of drugs and reported data on water monitoring.

## 2. Materials and Methods

### 2.1. Assessment of P&EDC of Concern in Brazilian Water

**Figure 1:**
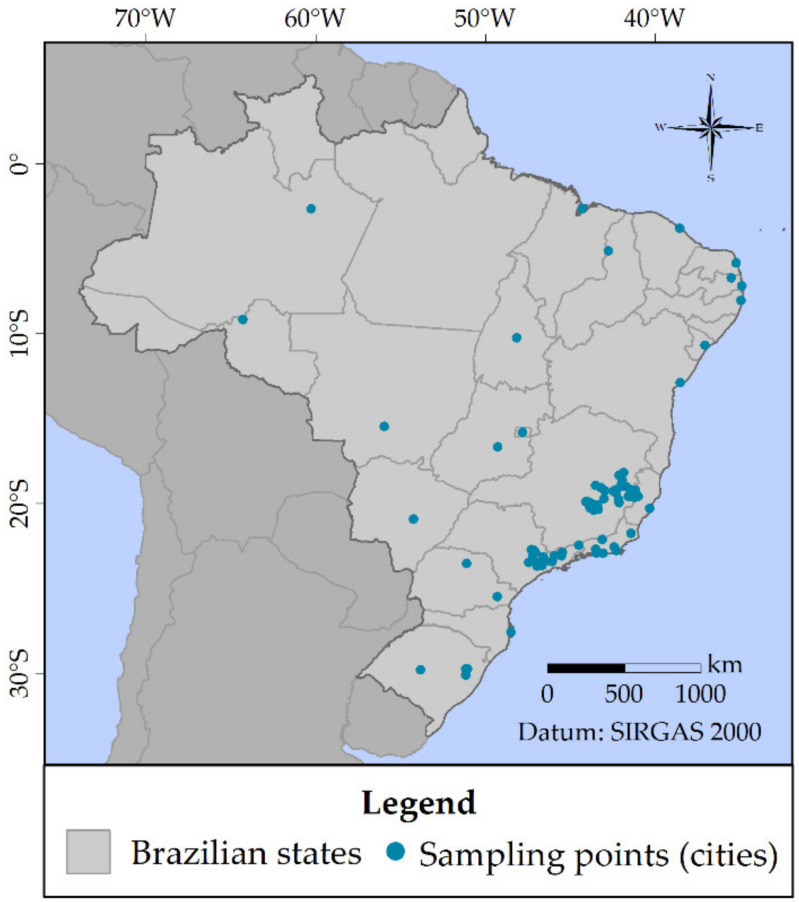
Sampling sites of the monitoring studies surveyed to compile data on occurrence of P&EDC compounds in Brazilian natural and drinking water.

In this work, a P&EDC list was produced considering the following criteria:

- Data from the pharmaceutical market: pharmaceuticals listed in the Brazilian sales ranking were selected for the P&EDC list. The 20 top-selling pharmaceutical active ingredients and associations of active ingredients in Brazil have been used. These data were obtained from the “*2017 Pharmaceutical Market Statistical Yearbook*”, made available by the Brazilian Health Regulatory Agency (ANVISA) [52]. For the specific case of antibiotics, which have controlled sales, another data compilation reported by ANVISA was considered [53]. Thus, the six antibiotics most used in Brazil were also selected for the P&EDC list.
- Occurrence in surface, ground, and/or drinking water: by means of an exhaustive literature review, a P&EDC list was built up from compounds quantified in natural and/or drinking water of Brazil. Figure 1 depicts the sampling sites where such studies have been carried out. Furthermore, many foreign data were also collected to enable a comparison of values found in Brazilian studies.

### 2.2. Estimation of GV for P&EDC in Drinking Water

In this work, a quantitative chemical risk assessment (QCRA) approach was used for estimating GV for the selected P&EDC. In establishing GV or maximum acceptable values (MAV) for chemical substances in drinking water during a QCRA, tolerable risks are commonly assessed based on critical effects observed in dose-response studies in humans (epidemiological) or animals. This is usually calculated considering the highest dose in which no adverse effect is observed (NOAEL—no-observed-adverse-effect-level). Furthermore, such risk assessments also consider safety (or uncertainty) factors and the proportion of the acceptable daily intake that may be allocated to drinking water (allocation factor). For the case of compounds that are known to be or potentially carcinogenic to humans, GV or MAV are usually calculated by extrapolating dose-response studies so that ‘adequate doses’ can be chosen based on an additional cancer risk [33,35,51]. For pharmaceuticals, an additional approach based on the lowest daily therapeutic dose (LDTD) can also be used [33].

There are several pharmaceuticals in the Brazilian market and new products are continuously developed and released for sale. These compounds are extensively studied in terms of risks to human health. Pharmaceuticals are rigorously tested to ensure the safety of the intended use, and there are programs to report and monitor their adverse effects on human health [54,55]. However, several data are confidential and unavailable for establishing GV for drinking water. On the other hand, pharmacology and therapeutic doses are typically reported by pharmaceutical manufacturers or easily found in pharmacopoeias. In general, for most pharmaceuticals, the doses which may cause toxicity are higher than that used for therapeutic purposes [15]. In applying QCRA, several toxicological studies available in the literature on pharmaceuticals lead to GV for drinking water higher than its LDTD. In this sense, several authors use the LDTD as a reference to define QCRA of pharmaceuticals in drinking water [33,56,57,58,59,60,61].

In this work, GV for P&EDC have been estimated through Equations (1) and (2), considering different approaches for deriving reference doses (NOAEL; LOAEL—lowest-observed-adverse-effect-level; BMDL_10_ –lower limit of critical effect of the benchmark dose; slope factor; and/or LDTD). Given the Brazilian reality, it was assumed a population average body weight of 60 kg and an average daily water intake of 2 L.

(1) $$\mathrm{Guideline}\;\mathrm{value}\;(\mathrm{GV}\;-\;\mu g/L)=\frac{\mathrm{ADI}\;(\mu g/\mathrm{kg}/d)\;\times \;\mathrm{BW}(\mathrm{kg})\;\times \;\mathrm{AF}}{V(L/d)}$$

where: ADI—acceptable daily intake, BW—body weight (60 kg for the case of a Brazilian adult), AF—allocation factor (proportion of the ADI that may be attributed to drinking water consumption, variable as a function of the CEC), and V—average daily water intake (2 L/d for the case of the Brazilian population).

(2) $$\mathrm{Acceptable}\;\mathrm{daily}\;\mathrm{intake}\;(\mathrm{ADI}\;-\;\mu g/\mathrm{kg}/d)=\frac{\mathrm{Reference}\;\mathrm{dose}\;(\mu g/\mathrm{kg}/d)}{\mathrm{UF}}$$

where: Reference dose—LDTD, NOAEL, LOAEL, etc. UF—uncertainty factor.

The GV calculated through the LDTD are divided by an uncertainty factor which provides a reasonable assurance that pharmacological or toxic effects are improbable. In this paper, the approach described in the Australian Guidelines for Water Recycling—Augmentation of Drinking Water Supplies [33] was considered for obtaining uncertainty factors to be applied for LDTD-based GV. According to EPHC/NRMMC/AHMC [33], an uncertainty factor of 1000 may be applied for the derivation of GV from the LDTD. However, uncertainty factors lower (less conservative) than the EPHC/NRMMC/AHMC [33], ranging from 22 to 500, have also been reported in the literature [56,57,59,60].

The approach considered in this work for deriving GV from LDTD considered an UF of 1000 which was the product of the following:

(a) a factor of 10 to account for response changes within humans (intraspecies variation);
(b) a factor of 10 for the protection of sensitive subgroups as children and infants;
(c) a factor of 10 to account for the fact the LDTD is not a no-effect level, i.e., the approach is similar to applying LOAEL instead of NOAEL.

On the other hand, for deriving GV from NOAEL or LOAEL, the following uncertainty factors were considered:

(d) a factor of 10 to account for interspecies variations, considering the uncertainty of extrapolating data from studies on experimental animals to humans;
(e) a factor of 10 to account for intraspecies variations;
(f) a factor of 10 when using data from a subchronic study (in absence of a chronic study);
(g) a factor of 10 when using LOAEL instead of NOAEL.

In both approaches (NOAEL/LOAEL or LDTD-based GV), an additional safety factor of 10 was applied for the case of cytotoxic pharmaceuticals, considering the high toxicity level of these compounds. Furthermore, an additional safety factor of 10 was also applied for endocrine disruptors, considering that the potential effects on hormonal function and fertility are unwanted in those not being treated with these medications [33].

Different allocation factors were adopted depending on the authorized use of the pharmaceutical [33], as follows:

(a) an allocation factor of 1.0 was adopted for pharmaceuticals prescribed only to humans, based on the premise that such CEC are not widespread in the environment and, therefore, are unlikely to be found in food;
(b) an allocation factor of 10% of the acceptable daily intake to drinking water (AF = 0.1) was considered for the case of pharmaceuticals used for agricultural or veterinary purposes (some of these may also be prescribed to humans) [33];
(c) an allocation factor of 20% was used (AF = 0.2) for natural estrogen hormones and compounds that mimic them (nonylphenol and octylphenol). This is the same value applied by USEPA [35], which considers that this value may safely ensure ingestion from other sources (e.g., for the case of estrogens, from milk and derivates, meat, etc.).
(d) For bisphenol A, an allocation factor of 60% of the acceptable daily intake (AF = 0.6) was adopted based on a study completed by the European Food Safety Authority (EFSA). From extremely conservative estimates, EFSA [62] indicated that exposure to bisphenol A from diet and other sources (inhalation of dust, dermal contact through cosmetics, etc.) corresponds to approximately 1.35 µg/kg/d, or 34% of the acceptable daily intake of 4.0 µg/kg/d. Thus, it would be possible to admit an allocation factor to drinking water of up to 0.66 for this acceptable daily intake.

Additionally, GV for compounds known carcinogens to humans were calculated based on the risk of carcinogenicity, as given by Equation (3).

(3) $$\mathrm{Guideline}\;\mathrm{value}\;(\mathrm{GV}\;-\;\mu g/L)=\frac{R\;\times \;\mathrm{BW}(\mathrm{kg})}{\mathrm{SF}(\mathrm{kg}.d/\mathrm{mg})\;\times \;V(L/d)}\;\times \;\frac{1000\;(\mu g)}{1\;(\mathrm{mg})}$$

where: R—lifetime cancer risk (10^−4^ to 10^−6^), BW—body weight (60 kg for a typical Brazilian adult), SF—OEHHA Slope Factor, and V—average daily water intake (2 L/d for the case of the Brazilian population).

### 2.3. Exposure via Drinking Water Consumption

The population margin of exposure (ME) was estimated by Equation (4). This is the same approach adopted by the USEPA for establishing the fourth contaminant candidate list (CCL-4) [35] and in the Australian Guidelines for Water Recycling [33].

(4) $$\mathrm{Margin}\;\mathrm{of}\;\mathrm{exposure}\;(\mathrm{ME})=\frac{\mathrm{GV}\;(\mathrm{ng}/L)}{\mathrm{OC}\;(\mathrm{ng}/L)}$$

where: GV—Guideline value (the lowest guideline value estimated for a given CEC) and OC—Occurrence of a given CEC in drinking water (maximum concentration reported in literature or n-th percentile of monitoring data).

The USEPA adopts the 90th percentile of concentrations found in drinking water to obtain the factor “OC” (occurrence) used in Equation (4). In the impossibility of calculating the 90th percentile, USEPA adopts the maximum quantified concentration [35]. On the other hand, the Australian Guidelines for Water Recycling adopts the maximum concentrations quantified in drinking water [33]. In this work, due to an impossibility of calculating percentiles of literature data (in general, raw data are not available in publications), the maximum concentration reported was used to estimate the margin of exposure to P&EDC via drinking water.

The ME represents how much the occurrence of a contaminant is lower or higher than its GV, and may be interpreted as follows:

- ME ≤ 1: CEC found in drinking water at concentrations greater than or equal to the GV and which, therefore, may represent a high risk to human health;
- 1 < ME ≤ 10: compounds found in drinking water at concentrations slightly lower than the GV, which indicates an alert situation since the occurrence is at the same order of magnitude of the concentration that would represent health risks.
- 10 < ME ≤ 100: compounds that are found in drinking water at concentrations below GV up to 2 orders of magnitude, which might be classified as a moderate risk to human health;
- 100 < ME ≤ 1000: compounds that are found in drinking water at concentrations below GV up to 3 orders of magnitude, which might be classified as a low risk to human health;
- ME > 1000: compounds that are found in drinking water at concentrations well below GV (higher than 3 orders of magnitude), which might be classified as a negligible risk to human health.

### 2.4. Risk of Antibiotics in Inducing Antimicrobial Resistance

Predicted no effect concentrations (PNEC values) of antibiotics, as presented by Bengtsson-Palme and Larsson [63], were used here to estimate the risk quotient (RQ) of antimicrobial resistance induction in natural water according to Equation (5). For this, we used the maximum concentration of antibiotics observed in Brazilian raw water (surface water), as reported in Table 1, as the environmental concentration.

(5) $$\mathrm{Risk}\;\mathrm{Quotient}\;(\mathrm{RQ})=\frac{\mathrm{MEC}\;(\mathrm{ng}/L)}{\mathrm{PNEC}\;(\mathrm{ng}/L)}$$

where: MEC—maximum environmental concentration, PNEC—predicted no effect concentration of antibiotics regarding resistance selection.

According to Zhao et al. [64], RQ ≥ 1 indicates high ecological risk; 0.1 ≤ RQ < 1 indicates moderate risk, and RQ < 0.1 indicates low ecological risk regarding a considered endpoint. Although the induction of antimicrobial resistance in bacteria is not an ecotoxicological endpoint, it was considered here as an adverse effect, at least from the human point of view, and hence used to set the risk classification as proposed by Zhao et al. [64].

**Table 1:** Occurrence of pharmaceuticals and endocrine disrupting compounds (P&EDC) in Brazilian and foreign water (…).

Compound	Matrix	Brazil			Other Countries		
		N	Concentration Range (ng/L)	References	N	Concentration Range (ng/L)	References
Paracetamol/ Acetaminophen ^1^	RW	132	<0.20–2147.00	[31,65,66,67,68]	nr	<1.83–43.518	[1,69,70,71,72,73]
	DW	121	<0.20–453.60	[31,65,66,67,68]	nr	<LD ^a^–7.00	[69,74]
Loratadine ^2^	RW	105	<1.90–486.00	[31,65,66,67,68]	nr	<0.03–0.6	[70,73]
	DW	104	<1.90–67.00	[31,65,66,67,68]	-	-	-
Promethazine ^2^	RW	23	<0.30–77.40	[31,65,66,67,68]	1	<0.2	[71]
	DW	20	<0.30–30.84	[65,66,67,68]	-	-	-
Amoxicillin ^3^	RW	74	<0.46–8.90	[31,32]	31	<LD	[1]
	DW	72	<31.50	[32]	21	<LD	[74]
Cefalexin ^3^	RW	2	<0.64–29.00	[31]	nr	283	[73]
	DW	-	-	-	-	-	-
Ciprofloxacin ^3^	RW	2	<0.41–2.50	[31]	nr	<LD–1407	[1,72,73,75,76]
	DW	-	-	-	25	<20	[74,75]
Clarithromycin ^3^	RW	72	<63.50	[32]	nr	<1.22–9831.5	[1,69,70,72,73,76]
	DW	72	<32.50	[32]	nr	<LD	[69,74]
Enoxacin ^3^	RW	72	<134.00–386.00	[32]	nr	< 2250 ^b^	[77]
	DW	72	<401.60	[32]	-	-	-
Enrofloxacin ^3^	RW	72	<11.80–71.00	[32]	nr	<LD–142.3	[1,73,76]
	DW	72	<5.00–219.00	[32]	-	-	-
Linezolid ^3^	RW	21	<1.75	[65,66,67,68]	19	<LD–87.6	[72]
	DW	20	<1.75–901.20	[65,66,67,68]	-	-	-
Norfloxacin ^3^	RW	74	<0.40–285.00	[31,32]	nr	<6.64–261	[1,69,70,73,75,76]
	DW	72	<39.30–210.00	[32]	nr	< 20.00	[69,75]
Sulfamethoxazole ^3^	RW	74	<0.80–1826.30	[31,65,66,68]	nr	<0.25–1820	[1,58,69,70,71,72,73,75,76]
	DW	70	<1.10–2592.60	[31,65,66,67,68]	nr	<0.25–1.81	[58,69,73,74,75]
Tetracycline ^3^	RW	2	<2.50–11.00	[31]	21	< LD	[74]
	DW	-	-	-	-	-	-
Trimethoprim ^3^	RW	81	<0.60–1573.90	[31,32]	nr	<0.25–176	[1,58,69,72,75]
	DW	78	<0.60–4381.20	[31,32]	nr	<14	[58,69,75]
Metformin ^4^	RW	138	<1.39–203.00	[32,65,66,67,68]	nr	8.4–3200	[69,73]
	DW	138	<1.39–111.20	[32,65,66,67,68]	nr	<LD	[69]
Fluconazole ^5^	RW	72	<7.40–1413.00	[32]	58	<0.30–898.8	[70,72]
	DW	72	<8.70–750.00	[32]	-	-	-
Atenolol ^6^	RW	72	<20.50	[32]	nr	<0.25–941.1	[1,58,69,70,71,72,73,78,79]
	DW	72	<14.50	[32]	nr	<0.25–715.00	[58,73,74,78,79]
Diltiazem ^6^	RW	21	<1.22	[65,66,67,68]	nr	<LD–6.9	[73]
	DW	20	<1.22	[65,66,67,68]	-	-	-
Losartan ^6^	RW	66	<1.00–926.00	[65,66,67,68]	nr	<14.00–620.00	[69,70,72,73,78]
	DW	66	<1.00–576.40	[65,66,67,68]	nr	<14.00–150.00	[69,73,78]
Propanolol ^6^	RW	66	<8.30–271.20	[65,66,67,68]	85	<1.1–537	[1,78]
	DW	66	<8.30–6837.00	[65,66,67,68]	26	<1.1–130	[78]
Atorvastatin ^7^	RW	72	<80.80–1020.00	[32]	nr	<0.50–530	[1,58,69,71,73]
	DW	72	<25.30–654.00	[32]	nr	<0.50	[58,69,74]
Bezafibrate ^7^	RW	75	<71.70–1365.00	[31,65,66,67,68]	nr	<1.8–256.7	[1,69,70,73]
	DW	71	<2.90–1659.10	[31,65,66,67,68]	nr	<LD–0.16	[69,73,74]
Gemfibrozil ^7^	RW	202	<0.30–2032.00	[31,32,65,66,67,68]	nr	<0.25–210	[1,58,69,71,73,75]
	DW	193	<0.30–2253.00	[31,32,65,66,67,68]	nr	<0.25–300	[69,74,75]
Triclosan ^8^	RW	20	<0.70–66.00	[30,31]	nr	<1.0–102	[1,58,73,75]
	DW	100	<3.00	[30]	nr	<1.0–1.93	[58,73,75]
Cimetidine ^9^	RW	74	2.60–13.90	[31,32]	1	<1.8	[71]
	DW	72	<29.60	[32]	-	-	-
Omeprazole ^9^	RW	72	<32.00	[32]	nr	<2.96	[70,73]
	DW	72	<17.80	[32]	21	<LD ^a^	[74]
Ranitidine ^9^	RW	74	8.30–15.80	[32]	nr	<5.0–498	[1,71,73]
	DW	72	<26.70	[32]	-	-	-
Acyclovir ^10^	RW	21	<0.95–220.40	[65,66,67,68]	9	<10	[80]
	DW	20	<0.95–93.08	[65,66,67,68]	5	<10	[80]
Bisphenol A ^11^	RW	166	<0.03–64,831.00	[30,31,65,66,67,68]	nr	<0.99–763	[1,58,73,75,79]
	DW	227	<0.03–2549.10	[30,31,65,66,67,68]	nr	<0.99–683	[58,72,73,74,75,79]
4-Nonylphenol ^11^	RW	145	<0.10–1918.00	[30,31,65,66,67,68]	56	<2.05–130.00	[1,58,79]
	DW	224	<0.10–2820.00	[30,31,65,66,67,68]	27	<2.05–16	[74,79]
4-Octylphenol ^11^	RW	134	<0.10–835.10	[31,65,66,67,68]	nr	<0.66	[1,69,79]
	DW	123	<0.20–276.60	[31,65,66,67,68]	nr	<0.66	[69,74,79]
17-beta-Estradiol ^12^	RW	146	<0.25–6806.00	[31,65,66,67,68]	nr	<0.81–4.04	[1,73,79]
	DW	123	<0.25–43.50	[31,65,66,67,68]	nr	<0.81	[74,79]
Estriol ^12^	RW	138	<0.08–67.40	[30,31,65,66,67,68]	36	<4.70–72.00	[78]
	DW	221	<0.08–97.40	[30,31,65,66,67,68]	36	<4.70	[78]
Estrone ^12^	RW	143	<0.07–279.50	[30,31,65,66,67,68]	nr	<0.20–130	[1,58,69,78,79]
	DW	223	<0.07–94.80	[30,31,65,66,67,68]	nr	<0.92	[58,69,74,78,79]
17-alpha- Ethinylestradiol ^12^	RW	153	<0.39–4390.00	[30,31,65,66,67,68]	nr	<0.20–3.40	[1,73,78,79]
	DW	223	<0.39–623.00	[30,31,65,66,67,68]	nr	<2.66	[74,78,79]
Levonorgestrel ^13^	RW	9	<1.00–663.00	[30,31]	39	<0.42	[70]
	DW	100	<1.00	[30]	11	<0.05–0.7	[81]
Acetylsalicylic acid ^14^	RW	12	<0.041–15,687.90	[31]	nr	<LD–1130	[1,69,73]
	DW	6	<0.04–5286.90	[31]	nr	<LD	[69]
Diclofenac ^14^	RW	72	<0.28–723.20	[31,65,66,67,68]	nr	<0.25–10,200	[1,58,69,70,72,73,79,82]
	DW	59	<0.28–1405.00	[31,65,66,67,68]	nr	<0.25–2.37	[58,69,73,74,79]
Ibuprofen ^14^	RW	205	0.02–4155.50	[31,32,65,66,67,68]	nr	<1.96–17,600	[1,69,71,73,75,79]
	DW	195	<0.28–490.20	[31,32,65,66,67,68]	nr	<18.00	[69,73,74,75,79]
Ketoprofen ^14^	RW	72	<34.70–1020.00	[32]	nr	<LD–9220	[1,69,70,73]
	DW	72	<64.60–561.00	[32]	nr	<LD–40	[69,73,74]
Naproxen ^14^	RW	64	<0.20–22,408.00	[31,32,65,66,67,68]	nr	<0.50–59,300	[1,58,69,73]
	DW	53	<0.20–372.632.00	[32,65,66,67,68]	nr	<LD–3.12	[58,69,73,74]
Betamethasone ^15^	RW	72	<8.00–11,960.00	[32]	17	0.29–7.2	[83]
	DW	72	<8.00–2620.00	[32]	11	<0.02–1.0	[81]
Dexamethasone ^15^	RW	79	<2.86–2159.00	[65,66,67,68]	nr	<LD–9.7	[69,73,83]
	DW	78	<2.86–2271.00	[65,66,67,68]	nr	<0.02–< 0.05	[69,81]
Prednisone ^15^	RW	72	<5.10–8105.00	[32]	nr	<0.02–1.3	[69,83]
	DW	72	<4.8–6323.00	[32]	nr	<0.03–0.05	[69,81]

N: amount of data; RW: raw water (water source that feeds a WTP); DW: drinking water (water distributed by a WTP); nr: not reported in the original paper; LD: limit of detection; ^a^: data not reported; ^b^: maximum. ^1^: analgesic/antipyretic; ^2^: antiallergic; ^3^: antibiotic; ^4^: antidiabetic; ^5^: antifungal; ^6^: antihypertensive; ^7^: antilipemic; ^8^: antiseptic; ^9^: antiulcer; ^10^: antiviral; ^11^: chemical input; ^12^: estrogen; ^13^: hormonal contraceptive; ^14^: nonsteroidal anti-inflammatory; ^15^: steroidal anti-inflammatory. Raw data can be found in Tables S2 and S3.

## 3. Results

### 3.1. Assessment of P&EDC of Concern in Brazilian Water

Table S1 (Supplementary Materials) shows the ranking of the top-selling pharmaceuticals active ingredients and associations of active ingredients in Brazil. Three of the best seller pharmaceuticals in Brazil (losartan potassium, metformin hydrochloridrate, and hydrochlorothiazide) are active ingredients prescribed for controlling hypertension and blood glucose, which are, respectively, the main causes of heart diseases and diabetes. However, the data from Brazilian pharmaceutical market do not represent the total mass/volume of active ingredient sold (e.g., tons of active ingredient), but comprise the number of sold units of pharmaceuticals containing a given active ingredient. Thus, as the dosages of active ingredients may vary between medications, data from the pharmaceutical market should be used with caution and only as additional information for defining priority P&EDC for risk assessment. It is also important to point out that several pharmaceuticals are distributed free of charge by the Brazilian Unified Health System (SUS) and, hence, are not reported in the sales ranking. In addition to the pharmaceuticals listed in Table S1, the antibiotics amoxicillin, cefalexin, azithromycin, trimethoprim, sulfamethoxazole, and tetracycline, which are the most used in Brazil, are also included in the list of compounds selected for risk assessment. According to Castro et al. [53], these antibiotics represented 96.5% of sales of the 10 most consumed antibiotics between the years 2013 and 2016.

Table 1 shows the ranges of occurrence of P&EDC monitored in Brazilian ground and/or drinking water and its comparison to many studies performed in other countries. Even though albendazole, azithromycin, amlodipine besilate, clonazepam, dipyrone, enalapril, hydrochlorothiazide, levothyroxine, nimesulide, sildenafil, and simvastatin were listed as top-selling pharmaceuticals, no studies were found on their occurrence in Brazilian water. On the other hand, there are also many pharmaceuticals monitored in Brazil that were not listed in the sales ranking. Furthermore, there are some pharmaceuticals listed as best sellers, such as atenolol, clarithromycin, and omeprazole, that were not detected/quantified in raw and/or drinking water in Brazil.

Data shown in Table 1 evidenced a great diversity of compounds present in surface and drinking water monitored in Brazil, as well as the amplitude of their concentrations. The preference of Brazilian researchers for monitoring antibiotics and hormones follows a world trend and is supported by the environmental impact of these compounds. Estrogenic hormones are environmentally relevant since they may cause feminization of some aquatic species, a decrease in sperm counts, as well as breast, uterine, and testicular cancer amongst others adverse effects [84]. In addition to causing biological toxicity and genotoxicity, antibiotics induce antimicrobial resistance in pathogenic bacteria, which is regarded by the World Health Organization as a global health and development threat [85,86,87].

It is seen in Table 1 that bisphenol-A, 17-beta-estradiol, 17-alpha-ethinylestradiol, and 4-nonylphenol are the endocrine disruptors detected in higher concentrations in Brazilian water sources with maximum concentrations of 64,831 ng/L, 6806 ng/L, 4390 ng/L, and 1918 ng/L, respectively. In drinking water, these compounds were also found in relatively high levels (maximum concentrations of 2549 n/L, 44 ng/L, 623 ng/L, and 2820 ng/L, respectively). In general, maximum quantified concentrations of estrogens and xenoestrogens found were lower in drinking water than in raw water. It is important to point out that these maximum concentrations are derived from different studies carried out in the same region (Campinas—São Paulo) [23,26,88] where these P&EDC were found in much higher concentrations than other locations (Tables S2 and S3 in Supplementary Materials).

The pharmaceuticals naproxen, acetylsalicylic acid, betamethasone, prednisone, and ibuprofen stood out for being detected in the highest concentrations among the compounds analyzed in Brazilian water sources (maximum values equal to 22,408.0 ng/L, 15,687.9 ng/L, 11,960.0 ng/L, 8105.0 ng/L, and 4155.5 ng/L, respectively), as seen in Table 1. Regarding the antibiotics monitored in drinking water in Brazil, the occurrence of trimethoprim (maximum concentration of 4381.2 ng/L) and sulfamethoxazole (maximum concentration of 2592.6 ng/L) were also noteworthy. As warned by different researchers [89,90], the presence of antibiotics in treated water may induce resistance in bacteria that grow in the distribution system which can, depending on residual chlorine levels and contact time, contribute to human exposure to resistance genes (ARG) and resistant bacteria (ARB).

In general, the less consolidated sanitation structure in Brazil seems to explain the fact that the concentrations of P&EDC quantified in Brazilian water sources are generally higher than those reported in developed countries. In the same way, the more incremented treatment used in the developed world, where the most of studies presented in this work (Tables S2 and S3) were done, may explain the tendency of lowest P&EDC concentration in treated water in such countries. In addition, the highest concentrations of P&EDC have been observed in Brazilian regions with high population density and during dry periods [31].

Once released into a water body, the concentration of a given P&EDC may be reduced by natural mechanisms, such as hydrolysis (nonredox reaction with water), volatilization (passage to the gas phase), adsorption (retention on the surface of solids), absorption (encapsulation, for example, by oil droplets), redox reactions (reaction between species with high reduction or oxidation potential), or photolysis (degradation by solar radiation) and biodegradation. Thus, analyzing the fate of a P&EDC in a water environment involves knowledge of its main characteristics and physicochemical properties, and this is decisive in predicting P&EDC occurrence in raw water. On the other hand, the prediction of P&EDC behavior during drinking water treatment is complex since it depends not only on its physicochemical properties but also on the technology used by the water treatment plant.

Some compounds found in high concentrations in Brazilian water, such as bisphenol A, 17-beta-estradiol, 17-alpha-ethinylestradiol, and nonylphenol, are hydrophobic (log Kow > 3.5), have low water solubility, and tend to remain attached to organic particles. In this sense, these results may seem to be contradictory and not consistent with environmental fate prediction. However, the presence of surfactants in Brazilian surface waters, mainly caused by the discharge or raw or partially treated sewage, may increase the solubility of such compounds and might explain a greater dispersion of these endocrine disruptors in the water bodies.

A list of P&EDC selected for further risk assessment is shown in Table 2 and was defined based on available data on Brazilian pharmaceutical market as well as published data of their monitoring in Brazilian water (raw and drinking water). Naphazoline was not included in the list because it is a nasal decongestant and the approach of using the LDTD cannot be applied for this drug since it is not administered orally.

### 3.2. Estimation of GV for Selected P&EDC in Drinking Water

Supplementary Table S4 shows a compilation of toxicological studies developed for the selected P&EDC regarded of priority concern in Brazil for the reasons exposed before (see Table 2). Values of acceptable daily intake were calculated from experimental doses (NOAEL, LOAEL or congeners, or even the LDTD for pharmaceuticals and hormones). Thus, corresponding concentrations in drinking water (guideline values—GV) were obtained from the acceptable daily intake considering the Brazilian reality. Due to this, values shown in Supplementary Table S4 differ from those reported in the original references.

According to IARC [91], 17-beta-estradiol is classified in Group 1 (carcinogenic to humans) with a slope factor of 39 kg.d/mg. In this sense, considering the carcinogenicity of 17-beta-estradiol, GV for the Brazilian reality would be between 0.0008 µg/L (risk of 10^−6^) and 0.08 µg/L (risk of 10^−4^). Adopting an additional cancer risk of 10^−5^, which is traditionally used [51], then a GV of 0.008 µg/L (8 ng/L) is obtained from Equation (3). This value is far below the GV of 0.30 µg/L (300 ng/L) shown in Supplementary Table S4, which was based on studies that assessed noncarcinogenic adverse effects. It is also important to consider that, for establishing the CCL-4 (Contaminant Candidate List), USEPA reports a GV of 0.0009 µg/L (0.9 ng/L) for 17-beta-estradiol. This value was obtained considering an additional cancer risk of 10^−6^, average body weight of 70 kg, and average daily water intake of 2 L [35].

One of the approaches used in deriving GV for safe exposure via water intake for natural and synthetic hormones (estrone, estriol, and 17-alpha-ethinylestradiol) was LDTD, as reported in the Australian Guidelines for Water Recycling [33]. However, despite the merits of this approach in the case of pharmaceuticals, there is no consensus regarding the side effects of any dose in hormone therapies [58,92,93]. For instance, IARC classifies combined oral contraceptives (estrogens with progesterone) as carcinogens to humans (group 1) [91] and considers 17-alpha-ethinylestradiol as representative of such contraceptive mixtures. There are also epidemiological studies that relate breast, cervical, and liver cancers in women and the combined hormone therapy using 17-alpha-ethinylestradiol [94]. Thus, in this work, acceptable daily intake and GV resulting from LDTD were not considered for the purposes of toxicity assessment and calculation of the margin of exposure of the Brazilian population to estrone, estriol, and 17-alpha-ethinylestradiol. Nonetheless, such values have been presented in Supplementary Table S4 for the sake of comparison. Exceptionally, acceptable daily intake and GV based on LDTD were considered for assessing margin of exposure for levonorgestrel and levothyroxine, due to a lack of toxicological or epidemiological studies for these hormones.

Table 3 shows a toxicity ranking with the compounds reported in Supplementary Table S4, based on the acceptable daily intake and GV estimated in the present work. As shown in Table 3, natural (estrone, 17-beta-estradiol, and estriol) and synthetic hormones (17-alpha-ethinylestradiol, levothyroxine, and levonorgestrel) exhibit high toxicity. On the other hand, the toxicity of most pharmaceuticals seems to be far lower than that of estrogen hormones. However, the anti-inflammatory corticosteroids betamethasone, dexamethasone, and prednisone, as well as the anxiolytic clonazepam, and the antihypertensives/antilipemics enalapril, amlodipine besilate, and simvastatin had toxicity in the same order of magnitude as the hormones.

### 3.3. P&EDC Exposure via Drinking Water Consumption

**Figure 2:**
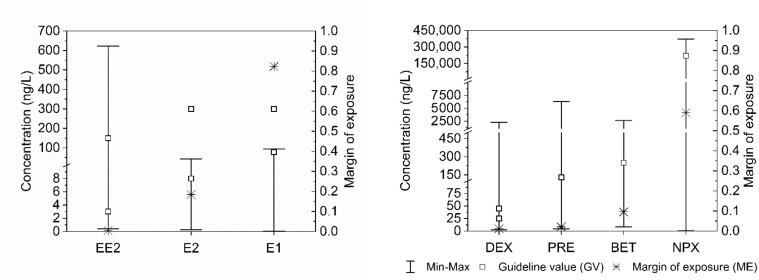
Concentration range, guideline values, and margin of exposure of pharmaceutical and endocrine disrupting compounds (P&EDC) quantified in Brazilian drinking water, which exhibited high risk. EE2 = 17-alpha-ethinylestradiol; E2 = 17-beta-estradiol; E1 = Estrone; DEX = Dexamethasone; PRE = Prednisone; BET = Betamethasone; NPX = Naproxen.

Supplementary Table S5 shows the margin of exposure of P&EDC detected in Brazilian drinking water and its level of risk. Of the 55 compounds listed in Table 2, it was possible to calculate the exposure margin for 41 of them, which had data on occurrence in Brazilian treated water. The margin of exposure (ME) was higher than 100, thereby setting the risk as low (10 compounds) or negligible (8 compounds) for 44% of these. For nine compounds the risk was deemed moderate (10 < ME < 100), whereas for seven compounds there was an alert situation (1 < ME < 10), since the compounds occurred in treated water in concentration slightly higher than the estimated threshold values. Finally, for seven compounds (the estrogen hormones 17-alpha-ethinylestradiol, 17-beta-estradiol, and the anti-inflammatories betamethasone, dexamethasone, prednisone, and naproxen) the risk was considered high since the estimated margin of exposure was lower than 1 (Figure 2).

The values of margin of exposure were calculated considering the maximum concentration quantified in drinking water, and this is excessively conservative. Considering that there are few studies on the occurrence of CEC in natural and drinking water in Brazil and, consequently, there is a small number of monitoring data, the maximum values or even the 95th percentiles often represent extreme concentrations which might be punctually seen in a study or location (outliers). In most cases, for compounds for which there is a more robust database, these extreme values do not represent the reality of the country. This highlights the importance of a nationwide program for monitoring water quality to verify the occurrence of CEC in water taken for public supply. The adoption of ‘contaminants candidate lists’ or ‘watch lists’ by some developed countries seems to be a good example to be followed.

Median concentrations of 17-alpha-ethinylestradiol, 17-beta-estradiol, and estrone hormones in Brazilian drinking water reported in the literature (<0.6 ng/L, <1.3 ng/L, and <1.8 ng/L, respectively [65]) were 10 to 100 times lower than GV based on toxicological/epidemiological studies (Table 3). According to Brandt et al. [65], estrone had the highest frequency of detection/quantification in raw and drinking water samples (27% and 28%) among these three hormones. However, estrone has lower estrogenic capacity than 17-alpha-ethinylestradiol and 17-beta-estradiol. Furthermore, only 1% of Brazilian drinking water samples had a concentration of estrone (94.8 ng/L) above the calculated GV of 78 ng/L (Table 2 and Table 3; Figure 2).

On the other hand, 17-alpha-ethinylestradiol was rarely detected/quantified in raw and drinking water (approximately 0% and 2.5%), despite its higher estrogenic capacity than estrone and 17-beta-estradiol. The maximum concentration of 17-alpha-ethinylestradiol (2.68 ng/L) and 17-beta-estradiol (4.30 ng/L) reported by Brandt et al. [65] remained below the estimated GV (Table 3). However, Lima et al. [31] reported a study carried out by Dias [29] in water treatment systems from three capitals in southeastern Brazil, in which the maximum concentrations of 17-alpha-ethinylestradiol (EE2) and 17-beta-estradiol (E2) in treated water were, respectively 236.6 ng/L and 43.4 ng/L. In that study, the median and/or average values in the treated water varied from 4.3 to 11.5 ng/L for E2 and from 3.7 to 155.7 ng/L for EE2. These values are in the same order of magnitude as the estimated GV presented in Table 3 for E2 (8 to 300 ng/L) and EE2 (3 to 150 ng/L), which led to the calculation of margins of exposure lower than unity for these compounds (Supplementary Table S5; Figure 2). For the case of estrone (E1), the maximum (70.1 ng/L) and the average (12,3 ng/L) concentrations in Brazilian drinking water reported by Lima et al. [31] remained below the lowest GV (78 ng/L), although it was detected in drinking water in one sample at a concentration of 94.8 ng/L in the state of Minas Gerais [65,68]. It is worth remembering that 17-beta-estradiol is proven to be carcinogenic in humans [91], and that estrone is a byproduct of estradiol degradation. Therefore, attention should be paid to such estrogenic compounds in polluted water intended for public supply.

Dexamethasone has been monitored in several large cities of Brazil [65,66,67,68]. However, it was detected/quantified in drinking water only in the state of Paraná (in 31% of the samples), corresponding to 5% of all samples. The mean concentration of dexamethasone in Brazilian drinking water (89.38 ng/L) reported by Brandt et al. [65] exceeded the GV of 25 ng/L estimated in the present work (Table 3, Figure 2). However, the median concentration reported by Brandt et al. [65] was lower than the LD (2.86 ng/L), which demonstrates the influence of extreme concentrations occasionally observed in the state of Paraná on the national median.

The low margin of exposure values estimated for prednisone and betamethasone (Figure 2) have drawn attention to these corticosteroid anti-inflammatories, which were found in drinking water at concentrations only one order of magnitude higher the GV (Table 2 and Table 3; Figure 2). However, these compounds were reported only in one Brazilian study [32], which evidences the need for more monitoring studies to confirm these results. In this single study, betamethasone was detected/quantified in 18% of drinking water samples at a median concentration lower than the LQ (8 ng/L), a value far lower than the GV of 250 ng/L estimated in this work (Table 3). The same situation was verified for prednisone. In the previously mentioned study, prednisone was detected/quantified in 32% of the samples collected in drinking water with a median concentration lower than the LQ (8 ng/L), which was also lower than the estimated GV (130 ng /L) (Table 3).

The detection of naproxen in Brazilian drinking water was reported by Brandt et al. [65], Teixeira et al. [66], Fazolo et al. [67], and Alves et al. [68]. Mean (8.091.0 ng/L) and median (48.3 ng/L) concentrations reported by Brandt et al. [65] were generally lower than the GV calculated in this work (220,000 ng/L) (Table 3, Figure 2), except for an extreme value (372,632 ng/L) (Table 2) observed in Rio Grande do Norte state.

In a report published by the Global Water Research Coalition [95], which synthesized the results of nine studies on the occurrence of pharmaceuticals in water supply systems, it was concluded that there was no risk to human health due to exposure to pharmaceuticals via drinking water intake. For comparison purposes, the study highlights that, if a person consumes drinking water throughout life containing pharmaceuticals at the levels normally found, the intake would be equivalent to less than 5% of a single daily therapeutic dose (equivalent to one medication pill). Likewise, one of the conclusions of another study entitled “Pharmaceuticals in Drinking Water” by the World Health Organization [15] is that most pharmaceuticals occur in natural and drinking water at concentrations below the therapeutic dose and, therefore, it is unlikely that the ingestion of water containing the concentrations usually found causes some adverse effect on human health. However, this does not mean that such pharmaceuticals do not cause adverse effects on the environment, particularly on aquatic species. This should be further explored in Brazil since the adoption of environmental standards of P&EDC to protect aquatic species might indirectly protect humans.

As stated at beginning of this section, the concentrations of pharmaceuticals listed in Table 3 that would cause chronic toxicity (GV) generally remained in the order of tens to thousands of times higher than the concentrations found in drinking water of Brazil. However, it is important to highlight the occurrence of endocrine disruptors. Although the concentrations of most endocrine disruptors remained below the reference values, estrogenic and xenoestrogenic compounds such as estrone, 17-beta-estradiol, estriol, 17-alpha-ethinylestradiol, bisphenol A and alkylphenols (e.g., nonylphenol and octylphenol) may affect many tissues and physiological functions of humans and other animals and mimic sex steroid hormones. As mentioned before, EE2, 4NP, and BPA are on the USEPA CCL-5 draft and BPA has been included in the European Union Drinking Water Directive with a GV (2.5 μg/L) much lower than estimated here (from 72 to 900 μg/L) from studies carried out with rats (see Table S4).

In spite of this, it is still worth remembering that a great safety margin—that generally reduced the critical effect dose by 1000 to 100,000 times due to the adoption of uncertainty (from 1000 to 10,000) and allocation (from 0.1 to 1) factors—was adopted here, which can reduce the calculated GV and enhance the estimated risks.

### 3.4. Antibiotic Resistance Induction in Raw Water

Some recent publications on the occurrence of resistant bacteria in several Brazilian surface waters are presented in Table 4. Several genera of antimicrobial resistant bacteria (ARB), some of them listed in the WHO priority list of microorganisms [48] have been detected in Brazilian surface waters around the country.

The most frequent bacterial group investigated in Brazilian surface waters was Enterobacteriaceae, either in terms of family or specific genera. This is an important group since species of this family including *Klebsiella pneumonia*, *Escherichia coli*, *Enterobacter* spp., *Serratia* spp., *Proteus* spp., *Providencia* spp., and *Morganella* spp. are resistant to carbapenem, cephalosporin, and third generation cephalosporin; hence, they are considered high priority pathogens by WHO [48]. *E. coli* is frequently evaluated in terms of its resistance to antibiotics in water environments, since it is part of the intestinal microbiota of several vertebrates, which are considered relevant reservoirs, epidemiologically connected or not. In addition, *E. coli* is released into the environment through fecal material, being regarded as the best fecal contamination indicator.

A study developed by Rebello and Regua-Mangia [96] demonstrated the circulation of *E. coli* isolates in the surface water of diverse urban and rural aquatic systems in Rio de Janeiro (RJ). These isolates exhibited resistance to a wide range of antibiotics, with higher percentages for cefalexin, and most of them also carried diarrheagenic genetic marker. *E. coli* resistant to amoxicillin, norfloxacin, ciprofloxacin, doxycycline, and sulfamethoxazole; strains producing β-lactamase with extended spectrum (ESBL) were also found among isolates from the Belém and Barigui rivers in Paraná [98]. A positive correlation between the number of Enterobacteriaceae isolates and the resistance genes abundancy was observed by Faria et al. [107] in a zoo lake in Cuiabá, indicating that urban sewage or another wastewater source was related to an increase in the amount of antibiotic-resistance genes in the environment.

As previous stated, several bacteria may exchange genes through horizontal gene transfer in the environment, which is one of the main mechanisms used to spread resistance traits. Integrons are mobile genetic elements that favor such transference and their presence may reveal a risk of antibiotic gene dissemination. For instance, in a study carried out by Canal et al. [97] most of the *E. coli* isolates from Patos Lagoon, located at Rio Grande do Sul, were considered resistant to quaternary ammonium compounds and all of them carried the qacE1 gene (a gene related to quaternary ammonium resistance) at the third conserved segment of the integron. The presence of both genes shows there is a risk of this microorganism to transfer these elements to other bacteria capable of infecting humans.

Despite *E. coli* being the most investigated species, it seems that less than a half of the isolates demonstrated an antibiotic resistance profile (Table 4). On the other hand, surface water isolates from *Aeromonas*, *Pseudomonas*, and *Staphylococcus genera* were highly resistant. Alves et al. [104] isolated cefotaxime and imipenem resistant bacteria in the Bolonha reservoir, which supplies water for the major water treatment plant of Belem (PA), in the Amazon region. In total, 98 bacterial isolates were obtained belonging to the genera *Pseudomonas* (37) and *Acinetobacter* (32) among others less frequent members of the Enterobacteriaceae family. These results stand out since species of these two genera are resistant to carbapenem and, hence, are considered as priority one by the WHO [48].

In addition to investigating ARB, Alves et al. [104] also assessed the presence of resistance genes (ARG) in the samples collected from the Bolonha reservoir. ARG were investigated for all isolates, and the most abundant were the β-lactams resistance genes blaCTX–M (28.3%), blaSHV (22.6%), and blaTEM (18.8%) in isolates from cefotaxime-supplemented medium and blaVIM (28.8%) and blaIMP (22.2%) in isolates recovered from imipenem-supplemented medium. In the same study, the resistome analysis approach confirmed that genes that confer resistance to β-lactams prevailed at all sampling sites, including enzymes that confer resistance to penicillins as well as to 1st- and 2nd-generation cephalosporins and also to extended-spectrum β-lactamases (ESBLs) of high clinical relevance. After β-lactams, the most frequent genes were those conferring resistance to aminoglycosides and tetracycline. Alves et al. [104] also identified genes associated with the virulence and defense subsystem, which have the potential to confer resistance to more than one class of antibiotics, as seems to be the case for genes encoding efflux pumps.

Considered priority two by the WHO [48], antibiotic-resistant *Staphylococcus* sp. and *Pseudomonas* sp. have been also detected in Brazilian surface waters. Studies on antibiotic resistance in Staphylococci isolated from the Dilúvio stream in southern Brazil revealed that 37% was resistant to erythromycin, 27% to penicillin, 12% to clindamycin, 6.8% to trimethoprim-sulfamethoxazole, 5% to chloramphenicol, and 2% to norfloxacin. In addition, 43% of Staphylococci strains were positive to one or more enterotoxin genes, therefore potentially pathogenic [100]. In another study, a total of 23 *Pseudomonas* sp. isolates were obtained and identified as *P. saponiphila*, *P. hunanensis*, *P. aeruginosa*, and *P. asiatica* in water samples from different cities of São Paulo state [102]. By using the Antimicrobial Susceptibility Testing, it was observed that these isolates had high minimum inhibitory concentration (MIC) to the tested antimicrobials (piperacillin-tazobactam, ceftazidime, ceftriaxone, cefotaxime, cefepime, aztreonam, imipenem, meropenem, gentamicin, tobramycin, tetracycline, ciprofloxacin, levofloxacin, norfloxacin, and chloramphenicol) and heavy metals (cadmium, cobalt, copper, mercury, and zinc) with the majority (*n* = 21; 91%) classified as multidrug-resistant (MDR) [102]. On the other hand, no *P. aeruginosa* resistant strains were observed in the Belém and Barigui rivers in Curitiba—PR [98].

Despite not being on the WHO’s priority list [48], *Aeromonas* has also been a concern regarding Brazilian drinking water quality. For instance, *Aeromonas* isolates from tap, mineral, and artesian well water were investigated revealing that the majority were multidrug resistant. The most active antimicrobial was ciprofloxacin (susceptible in 100% of the isolates), and the least active antibiotic was ampicillin (resistance in 91% of the isolates). Resistance to three or more antibiotics was frequently observed amongst isolates from environmental strains, even though it was not detected among clinical strains [103]. It is noteworthy that in the study all isolates grew after chlorine exposition of up to 1.2 mg/L during 1 min of contact [103]. According to the authors, it is not clear whether the higher tolerance to chlorine of *Aeromonas* isolates can be linked to a greater virulence.

In addition to the spread of resistant-fecal bacteria and their genes, the presence of the antibiotics themselves in the environment could confer a selective pressure to enrich ARB and therefore ARG. As discussed in the previous section, the antibiotic concentration in Brazilian water bodies is usually low, in the range of ng/L, which makes their quantification more difficult and restricted to few laboratories. Indeed, only a handful of papers have investigated the simultaneous presence of antibiotics, ARB, and/or ARG in water samples. One study carried out by Arsand et al. [106] in the Diluvio River, Porto Alegre (RS), found (in a range of <LQ to 344 ng/L) only 8 (azithromycin, cephalexin, ciprofloxacin, clindamycin, norfloxacin, sulfadiazine, sulfamethoxazole, and trimethoprim) out of 40 antibiotics surveyed in the collected water samples. Particularly, the quinolone class was highly detected, reaching values up to 650 ng/L in total. Four ARG conferring resistance to sulfonamides (sulI), β-lactams (blaTEM), erythromycin (ermB), and quinolones (qnrS) were also analyzed in the same water samples. Quantitative PCR analysis revealed the presence of ARG in all samples, with the highest concentration found for blaTEM. By comparing the concentration of antibiotics and the absolute abundance of ARG, it was possible to observe, for some samples, a congruence between blaTEM and qnrS genes and their target antibiotics. The decreased concentration of quinolones (ciprofloxacin and norfloxacin) was accompanied by the decrease in the absolute abundance of qnrS, and the same tendency was observed for cephalexin and blaTEM [106]. In another study with water samples from the Belém and Barigui rivers in Curitiba (PR), Böger et al. [98] observed antibiotics concentration in a range from 0.13 to 4.63 μg/L with amoxicillin being found in the highest concentration. The authors observed a correlation between antibiotic concentration and the rate of bacterial resistance, which was measured by antibiotic susceptibility tests from the coliforms isolates (*E. coli*, *Enterococcus* spp., and *P. aeruginosa*). These results suggest that the pollution of surface waters by anthropic activities influence antibiotic resistance [98].

It seems that antibiotic presence in the environment is not only related to an increase in the amounts of ARB or ARG but also to the development of more resistant ARB strains. This was demonstrated by Coutinho et al. [105] by analyzing the diversity of antibiotic resistant bacteria in aquatic environments subjected to distinct degrees of anthropogenic effects in Rio de Janeiro city (RJ). The authors observed that the microbial communities were capable of tolerating antibiotic concentrations up to 600 times higher than that used for clinical treatment. According to them, ampicillin-resistant bacteria were abundant and widespread in all impacted aquatic environments surveyed, except in Ilha Grande, a relatively well-preserved island located in the Atlantic rain forest biome, where no ampicillin-resistant bacteria was isolated.

All the studies mentioned so far were carried out in surface freshwater environments, some of them used as water sources to produce drinking water. There is not much information on ARB and ARG in drinking water or in water treatment plants in Brazil. One study carried out by Freitas et al. [99] isolated fecal coliforms resistant to antibiotics in drinking water and at a drinker surface. Strain identification revealed 31% of *Salmonella* spp. and 51% of other coliforms. Only a small fraction was resistant to the tested antibiotic (14.6% to ampicillin, 7.9% to tetracycline, and 3.4% to ciprofloxacin), but 27 strains had stable plasmids with the ability to perform conjugation and, hence, to disseminate ARG among other bacteria.

The maximum concentration of antibiotics found in Brazilian surface waters (see raw water, Table 1) has been used here to estimate the risk they pose on the induction of antimicrobial resistance, as predicted by Bengtsson-Palme and Larsson [63]. Table 5 shows the resulting risk classification for 10 antibiotics found in Brazilian water and for which there was a reported PNEC value. It is seen that for half of them the risk was deemed low, whereas for the other half it was moderate (30%) or high (20%).

Although the risk quotient for antimicrobial resistance induction is conservative since it employs the highest antibiotic concentration reported in Brazilian water, it is somehow worrying that these contaminants are in surface water in amounts that are in the same order of magnitude as the estimated PNEC values. This highlights the importance of collecting and treating sewage and to ensure that antibiotics are adequately removed during the biological treatment normally used for such.

Table 5 shows that for the antibiotics enrofloxacin and trimethoprim the risk quotient remained high even after the water treatment performed in the Brazilian water treatment plants surveyed. This brings attention to the fact that bacterial regrowth in the distribution system may lead to the development of biofilms, which are high bacterial density spots where induction of resistance may thrive. This may happen due to the presence of antibiotics or free resistance genes in the water, as implied by Bergeron et al. [108] who showed that water filtration was not efficient in retaining small fragments of bacterial DNA. In this regard, it is noteworthy to mention the study of O’Flaherty et al. [109] who examined the potential human exposure to antibiotic-resistant (AR) *Escherichia coli* through drinking water and found that a mean adult exposure to AR *E. coli* from tap water consumption ranged from 3.44 × 10^−7^ to 2.95 × 10^−1^ CFU/day depending mainly on the disinfection technology used for water treatment.

## 4. Conclusions

This work enabled the identification of 55 pharmaceutical and endocrine disrupting compounds (P&EDC) that are likely to be present in Brazilian surface waters used as sources for public supply. For 41 of these, there were data on occurrence in drinking water that enabled a quantitative chemical risk analysis (QCRA) through the estimation of their margin of exposure. Seven compounds (the anti-inflammatories betamethasone, dexamethasone, naproxen, and prednisone; and the estrogens 17-beta-estradiol, estrone, and 17-alpha-ethinylestradiol) stood out since their maximum reported concentration in treated water were higher than the threshold values estimated for an adverse effect to human health. Low guideline values were estimated for such compounds due to the lower values of acceptable daily intake reported in the literature (0.0001 to 0.05 µg/(kg_bw_.d). For these it is recommended further monitoring to confirm these findings and strengthen the yet scarce database on the occurrence of P&EDC in Brazilian waters. It is also seen that Brazilian surface waters contained antibiotics in concentrations allegedly able to induce antimicrobial resistance, especially for enrofloxacin and trimethoprim that showed the highest risk to induce resistance. The literature surveyed confirms that resistant bacteria (ARB) such as *Escherichia coli*, *Aeromonas* sp., *Pseudomonas* sp. and *Staphylococcus* sp., as well as several antibiotic resistance genes (ARG) are widely distributed in water sources around the country.

## Figures and Tables

**Table 2:** Pharmaceuticals and endocrine disrupting compounds (P&EDC) selected for further risk assessment.

Compound	Reason for Selection		Compound	Reason for Selection	
	Occurrence in Brazilian Water	Top-Selling in Brazil		Occurrence in Brazilian Water	Top-Selling in Brazil
Acyclovir	x		Gemfibrozil	x	
Acetylsalicylic acid	x		Hydrochlorothiazide		x
Albendazole		x	Ibuprofen	x	x
Amoxicillin		x	Ketoprofen	x	
Atenolol		x	Levonorgestrel	x	x
Atorvastatin	x		Levothyroxine		x
Azithromycin		x	Linezolid	x	
Amlodipine besilate		x	Loratadine	x	
Betamethasone	x		Losartan	x	x
Bezafibrate	x		Metformin	x	x
Bisphenol A	x		Naproxen	x	
Cefalexin	x	x	Nimesulide		x
Cimetidine	x		Nonylphenol	x	
Ciprofloxacin	x		Norfloxacin	x	
Clarithromycin		x	Octylphenol	x	
Clonazepam		x	Omeprazole		x
Dexamethasone	x		Paracetamol	x	x
Diclofenac	x	x	Prednisone	x	
Diltiazem	x		Promethazine	x	
Dipyrone		x	Propanolol	x	
Enalapril		x	Ranitidine	x	
Enoxacin	x		Sildenafil		x
Enrofloxacin	x		Simvastatin		x
17- beta-Estradiol	x		Sulfamethoxazole	x	x
Estriol	x		Tetracycline	x	x
Estrone	x		Triclosan	x	
17-alpha-Ethinylestradiol	x	x	Trimethoprim	x	x
Fluconazole	x				

**Table 3:** Toxicity ranking of pharmaceuticals and endocrine disrupting compounds (P&EDC) based on the lowest calculated values of acceptable daily intake (ADI) in comparison to the guideline value (GV).

	Compound	ADI (µg/kg bw/d)	GV (µg/L)		Compound	ADI (µg/kg bw/d)	GV (µg/L)
1°	17-alpha-Ethinylestradiol	0.0001–0.005	0.003 ^a^–0.15	29°	Trimethoprim	1.7–191	5.0–575
2°	Levothyroxine	0.0002	0.006	30°	Diltiazem	2.0	60
3°	17- beta-Estradiol	0.0003 ^b^–0.05	0.008–0.30	31°	Azithromycin	3.0–8.3	9.0–25
4°	Levonorgestrel	0.0005	0.015	32°	Cimetidine	3.3	100
5°	Dexamethasone	0.008	0.025–0.045	33°	Nimesulide	3.3	100
6°	Betamethasone	0.008	0.25	34°	Bisphenol A	4.0–50	72–900
7°	Clonazepam	0.008	0.25	35°	Paracetamol	5.4–50	160–1500
8°	Estrone	0.013–0.05	0.078 ^a^–0.30	36°	Enrofloxacin	6.2	20
9°	Enalapril	0.04–0.23	1.3–7.0	37°	Enoxacin	6.7	200
10°	Prednisone	0.042	0.13	38°	Albendazole	6.7	20
11°	Estriol	0.05	0.01–0.30	39°	Acetylsalicylic acid	7.2	22
12°	Amlodipine besilate	0.08	2.5	40°	Naproxen	7.3	220
13°	Simvastatin	0.08–0.7	2.5–20	41°	Metformin	8.3	250
14°	Loratadine	0.17	5.0	42°	Clarithromycin	8.3	250
15°	Atorvastatin	0.17–6.7	5.0–200	43°	Dipyrone	8.3–150	25–450
16°	Omeprazole	0.17	5.0	44°	Sulfamethoxazole	10–512	30–1535
17°	Hydrochlorothiazide	0.21	0.6	45°	Bezafibrate	10	300
18°	Sildenafil	0.21	6.3	46°	Cefalexin	10	30
19°	Promethazine	0.33	10	47°	Acyclovir	13.3	400
20°	Losartan	0.42	13	48°	Ibuprofen	13.3	400
21°	Diclofenac	0.5–67	1.5–200	49°	Linezolid	13.3	400
22°	Propanolol	0.5	15	50°	Norfloxacin	13.3	40
23°	Amoxicillin	0.5	1.5	51°	Octylphenol	15	90
24°	Fluconazole	0.8	25	52°	Nonylphenol	15–50	90–300
25°	Atenolol	0.8–2,7	25–80	53°	Gemfibrozil	20–31	600–930
26°	Ketoprofen	1.0–1.7	3.0–5.0	54°	Tetracycline	30	90
27°	Ranitidine	1.3	38	55°	Triclosan	50–75	150–225
28°	Ciprofloxacin	1.6–83	4.8–25				

^a^: For the case of 17-alpha-ethinylestradiol, 17-beta-estradiol, and estrone, the lowest therapeutic daily dose was not used to estimate the GV; ^b^: Considering carcinogenic effects.

**Table 4:** Reported occurrence of resistant bacteria (ARB) and resistance genes (ARG) in Brazilian surface waters (to be continued).

Target Organism ^1^	Location	Phenotypic Antibiotic Resistance (AR)	Genetic Elements	Reference
*Escherichia coli*	Surface water in agricultural area, Rio de Janeiro (RJ)	57.7% of isolates were resistant at least to one of the 11 antimicrobials tested: AMI, AMP, CFL, CPM, CFO, CIP, GEN, NIT, NOR, SUT, TRI.	nd	[96]
	Surface water in recreational area, Rio de Janeiro (RJ)	56.4% of isolates were resistant at least to one of the 11 antimicrobials tested: AMI, AMP, CFL, CPM, CFO, CIP, GEN, NIT, NOR, SUT, TRI.	nd	[96]
	Patos Lagoon (RS)	35% of isolates were resistant at least to one of the 17 antimicrobials tested: AMP, CFO, CPM, AMI, GEN, NOR, AMX/CLA, PIP/TZB, ATM, IPM, CAZ, CFT, CLO, TET, TRI/SMX, SUL, STM, SPM.	qacEΔ1, dfrA1, dfrA12, dfrA17, aadA1, aadA5, aadA22.	[97]
	Belém and Barigui Rivers, Curitiba (PR)	33% of isolates were resistant to AMX, 28% to SMX, 10% to NOR, 13% to CIP, 3% to DOX.	nd	[98]
Fecal coliforms	Drinking and surface water in Morrinhos (GO)	14.6% of the isolates were resistant to AMP, 7.9% to TET, 3.4% to CIP.	Stable plasmids	[99]
*Staphylococci*	Dilúvio River, Porto Alegre (RS)	37.50% of the isolates were resistant to ERI, 27.27% to PEN, 12.50% to CLI, 6.81% to TRI/SMX, 5.68% to CLO, 2.27% to NOR.	nd	[100]
*Enterococcus* spp.	Belém and Barigui Rivers, Curitiba (PR)	4% of isolates were resistant to CIP, 2% to NOR, 1.7% to VAN, none were resistant to AMX.	nd	[98]
	Rivers from Apucarana City, (PR)	One isolate was resistant to TET; any isolate was resistant to AMP, CIP, ERT, GEN, NOR, TET, VAN.	nd	[101]
*Pseudomonas*	Belém and Barigui Rivers, Curitiba (PR)	No resistance was observed among isolates.	nd	[98]
	Rivers, streams, and lakes from São Paulo	87% of the isolates were resistant to TET, 78% to CET, 78% to CFT, 74% to CLO, 62% to PIP/TZB, 61% to CAZ, 52% to ATM, 30% to CIP, 30% to LEV, 30% to NOR, 26% to CPM, 13% to IPM, 13% to MER, 9% to GEN and 9% to TBM.	blaGES, qnrS, qepA, tetB, aac(3′)-IIa, and ant(2″)-Ia, no plasmids were found.	[102]
*Aeromonas*	Mineral water, tap water, artesian water	91% of the isolates were resistant to AMP, 87% to CFL, 52% to CLO, 30% to CFT, 30% to ATM, 26% to GEN, 26% to TET, 26% to TMP/SMX, 17% to NAL and 4% to MER.	nd	[103]
Cefotazime resistant bacteria (*Acinetobacter*, *Pseudomonas*, *Klebsiella*, and *Enterobacter*	Bolonha reservoir, Pará	94.9% of isolates were resistant to three or more classes of antibiotics: 96.2% to ATM, 94.3% to CFT, 90.5% to AMX, 88.6% to AMP, 86.7% to NAL, 75.4% to CFL, 75.4% to CA and 71.6% to AMX/CLA.	blaCTX (28.3%), blaSHV (22.6%), blaTEM (18.8%), blaIMP (15.0%), blaVIM (3.7%).	[104]
Imipenen resistant bacteria (*Acinetobacter*, *Pseudomonas*, *Klebsiella*, and *Enterobacter*)	Bolonha reservoir, Pará	85.7% of isolates were resistant to three or more classes of antibiotics: 88.5% to AMX, 80.3% to ATM, 73.7% to AMP, 63.9% to IPM, 62.3% to CFL, 48% to KAN, 47.6% to NAL and 45.9% to CAZ.	blaVIM (28.8%), blaIMP (22.2%), blaCTX (8.8%), blaKPC (6.6%).	[104]
Ampicillin resistant bacteria (heterotrophs)	Parnaioca river, Rio de Janeiro	No ampicillin resistant bacteria were isolated from this site.	nd	[105]
Antibiotic resistance genes	Dilúvio River, Porto Alegre (RS)	nd	SulI: 10^1^–10^4^ gene copies/mL; blaTEM: 10^1^–10^6^ gene copies/mL; ermB: 10^0^–10^3^ gene copies/mL; qnrS: 10^1^–10^3^ gene copies/mL.	[106]
*Prevotella* spp., *Enterobacter* spp., *Aeromonas* *hydrophila*, *Staphylococcus epidermidis*, *Serratia* *marcescens*, *Erysipelothrix* spp., *Acinetobacter lwoffii,* and *Bacteroides fragilis*.	Uberabinha River, Uberlândia (MG)	nd	sul2, tetW, ermF blaIMP4, blaNDM1, intI2; tetB, tetC, tetM, and gyrA were dominant with an average level of 1.0 × 10^2^/16S rRNA copies.	[46]

^1^ Unless described, all isolates were obtained in growth media without a selective pressure of antibiotic; nd—not determined. Name abbreviations (antibiotic/gene): amikacin (AMI), aminoglycosides (aac(3′)-IIa, ant(2″)-Ia) amoxicillin (AMX), ampicillin (AMP), aztreonam (ATM), β-lactams (blaTEM, blaIMP4, blaGES and blaNDM1), cephalothin (CFL), cefepime (CPM), cefotaxime (CFT), cefoxitin (CFO), ceftazidime (CAZ), ceftriaxone (CET), ciprofloxacin (CIP), clavulanic acid (CLA), clindamycin (CLI), chloramphenicol (CLO), doxycycline (DOX), erythromycin (ERI), gentamicin (GEN), imipenem (IPM), integrons (intI1, intI2), kanamycin (KAN), levofloxacin (LEV), macrolides (ermB), meropenem (MER), nalidixic acid (NAL), nitrofurantoin (NIT), norfloxacin (NOR), penicillin (PEN), piperacillin (PIP), quinolones (qnrS, qepA, gyrA), sulfamethoxazole (SMX), sulfonamides (SUL/sulI), sulphazothrin (SUT), spectinomycin (SPM), streptomycin (STM), tazobactam (TZB), tetracyclines (TET/tetA, tetB, tetC, tetM, tetW), tobramycin (TBM), trimethoprim (TRI), and vancomycin (VAN).

**Table 5:** Risk estimation of antimicrobial resistance induction by antibiotics found in Brazilian surface waters.

	PNEC (μg/L) ^1^	PNEC (ng/L)	Occurrence in Raw (Surface) Water (ng/L) ^2^	MEC (ng/L)	RQ ^3^	Risk Classification
Amoxicillin	0.25	250	<0.46–8.9	8.9	0.03	Low
Cefaloxin	4	4000	<0.64–29	29	0.007	Low
Ciprofloxacin	0.064	64	<0.4–2.5	2.5	0.04	Low
Clarithromycin	0.25	250	<63.5	63.5	0.25	Moderate
Enoxacin	NA	NA	<134–386	386	NA	NA
Enrofloxacin	0.064	64	<11.8–71	71	1.11	High
Linezolide	8	8000	<1.75	1.75	0.0002	Low
Norfloxacin	0.5	500	<0.40–285	285	0.57	Moderate
Sulfamethoxazole	16		<0.8–1826.3	1826.3	0.11	Moderate
Tetracycline	1	1000	<2.5–11	11	0.01	Low
Trimethoprim	0.5	500	<0.6–1573.9	1573.9	3.15	High

PNEC: predicted no effect concentration of antibiotics regarding resistance selection; MEC: maximum environmental concentration; RQ: risk quotient; NA—not available; ^1^ As reported by Bengtsson-Palme and Larsson [63]; ^2^ As reported in Table 1; ^3^ According to Equation (5).

## Data Availability

Not applicable.

## Supplementary Materials

The following are available online at https://www.mdpi.com/article/10.3390/ijerph182211765/s1, Table S1: The 20 top-selling pharmaceutical substances in Brazil, by active ingredient or association of active ingredients. Table S2: Occurrence of Pharmaceuticals and Endocrine Disrupting Compounds (P&EDC) in Brazilian waters (raw data). Table S3: Occurrence of Pharmaceuticals and Endocrine Disrupting Compounds (P&EDC) in foreign waters (raw data). Table S4: Toxicological studies and guideline values (GV) for selected pharmaceutical and endocrine disrupting compounds (P&EDC). Table S5: Margin of exposure to contaminants of emerging concern (CEC) in Brazilian waters.

## Abbreviations

(aac(3′)-IIa, ant(2″)-Ia)	aminoglycosides resistance genes
ADI	acceptable daily intake
AF	allocation factor
AMI	amikacin
AMP	ampicillin
AMX	amoxicillin
APEO	alkylphenol ethoxylates
AR	antibiotic resistance
ARB	antibiotic resistant bacteria
ARG	antibiotic resistance genes
ATM	aztreonam
BET	Betamethasone
blaTEM, blaIMP4, blaGES and blaNDM1	β-lactams resistance genes
BMDL10	lower limit of critical effect of the benchmark dose
BPA	bisphenol A
CAZ	ceftazidime
CCL	contaminant candidate list
CEC	contaminants of emerging concern
CET	ceftriaxone
CFL	cephalothin
CFO	cefoxitin
CFT	cefotaxime
CFU	colony forming unit
CIP	ciprofloxacin
CLA	clavulanic acid
CLI	clindamycin
CLO	chloramphenicol
CPM	cefepime
DEX	dexamethasone
DOX	doxycycline
DW	drinking water (water distributed by a WTP)
E1	estrone
E2	β-Estradiol
E2 -17-beta-estradiol	
EE2	17-alpha-ethinylestradiol
EFSA	European Food Safety Authority
ERI	erythromycin
ermB	macrolides resistance gene
GEN	gentamicin
GV	guideline values
IARC	International Agency for Research on Cancer
intI1, intI2	integron genes
IPM	imipenem
KAN	kanamycin
LD	limit of detection
LQ	limit of quantification
LDTD	lowest daily therapeutic dose
LEV	levofloxacin
LOAEL	lowest observable adverse effect level
MAV	maximum acceptable values
ME	margin of exposure
MEC	maximum environmental concentration
MER	meropenem
NAL	nalidixic acid
NIT	nitrofurantoin
NOAEL	no observed adverse effect level
NOR	norfloxacin
NP	nonylphenol
NPX	naproxen
OC	occurrence concentration
PCR	polymerase chain reaction
P&EDC	pharmaceuticals and endocrine disrupting compounds
PEN	penicillin
PIP	piperacillin
PNEC	predicted no effect concentrations
PRE	prednisone
QCRA	Quantitative Chemical Risk Assessment
qnrS, qepA, gyrA	quinolone resistance genes
rRNA	ribossomal RNA
RQ	risk quotient
RW	raw water (water source that feeds a WTP)
SMX	sulfamethoxazole
SPM	spectinomycin
STM	streptomycin
SUL	sulfonamides
sulI	sulfonamide resistance gene
SUT	sulphazothrin
TBM	tobramycin
TET	tetracyclines
tetA, tetB, tetC, tetM, tetW	tetracycline resistance genes
TRI	trimethoprim
TZB	tazobactam
UF	uncertainty factor
USEPA	United States Environmental Protection Agency
VAN	vancomycin
WHO	World Health Organization
