# Supplementary material for: Occurrence of Pharmaceuticals and Endocrine Disrupting Compounds in Brazilian Water and the Risks They May Represent to Human Health
Source: Int J Environ Res Public Health. 2021 Nov 9;18(22):11765. doi: 10.3390/ijerph182211765 (PMC8620687; doi:10.3390/ijerph182211765)
Supplement: Supplementary file 1 [file ijerph-18-11765-s001.zip › ijerph-1403206-supplementary.pdf]

International Journal of
Environmental Research and Public Health

Article

Occurrence of Pharmaceuticals and Endocrine Disrupting Compounds in Brazilian Waters and the Risks They may Represent to Human Health

Sérgio Francisco de Aquino [1,*], Emanuel Manfred Freire Brandt [2], Sue Ellen Costa Bottrel [2], Fernanda Bento Rosa Gomes [3] and Silvana de Queiroz Silva [4]

[1] Chemistry Department, Federal University of Ouro Preto (UFOP), Ouro Preto, 35400-000, Brazil
[2] Environmental and Sanitary Engineering Department, Federal University of Juiz de Fora (UFJF), Juiz de Fora, 36036-900, Brazil; emanuel.brandt@ufjf.edu.br (E.M.F.B.); sue.bottrel@ufjf.edu.br (S.E.C.B.)
[3] Civil Engineering Graduate Programme, Federal University of Juiz de Fora (UFJF), Juiz de Fora, 36036-900, Brazil; fernanda.bento@engenharia.ufjf.br
[4] Biological Sciences Department, Federal University of Ouro Preto (UFOP), Ouro Preto, 35400-000, Brazil; silvana.silva@ufop.edu.br (S.Q.S)
* Correspondence: sergio@ufop.edu.br

Citation: Aquino, S.F.; Brandt, E.M.F.; Bottrel, S.E.C.; Gomes, F.B.R.; Silva, S.Q. Occurrence of Pharmaceuticals and Endocrine Disrupting Compounds in Brazilian Water and the Risks They May Represent to Human Health. *Int. J. Environ. Res. Public Health* **2021**, *18*, 11765. https://doi.org/10.3390/ijerph182211765

Academic Editor: Ivone Vaz-Moreira and Marcela França Dias

Received: 15 September 2021
Accepted: 5 November 2021
Published: 10 November 2021

Publisher's Note: MDPI stays neutral with regard to jurisdictional claims in published maps and institutional affiliations.

Copyright: © 2021 by the authors. Submitted for possible open access publication under the terms and conditions of the Creative Commons Attribution (CC BY) license (https://creativecommons.org/licenses/by/4.0/).

Table S1. The 20 top-selling pharmaceutical substances in Brazil, by active ingredient or association of active ingredients.

Ranking	Active ingredient	Pharmaceutical presentations sold
1º	Sodium chloride	More than 250 million
2º	Losartan potassium	Between 150 million and 250 million
3º	Dipyrone	Between 100 million and 150 million
4º	Metformin hydrochloridrate	Between 100 million and 150 million
5º	Hydrochlorothiazide	Between 50 million and 100 million
6º	Paracetamol	Between 50 million and 100 million
7º	Ibuprofen	Between 50 million and 100 million
8º	Levonorgestrel; ethinylestradiol	Between 50 million and 100 million
9º	Levothyroxine sodium	Between 50 million and 100 million
10º	Nimesulide	Between 50 million and 100 million
11º	Naphazoline hydrochloride	Between 50 million and 100 million
12º	Atenolol	Between 50 million and 100 million
13º	Sildenafil citrate	Between 50 million and 100 million
14º	Enalapril maleate	Between 25 million and 50 million
15º	Paracetamol; Diclofenac sodium	Between 25 million and 50 million
16º	Simvastatin	Between 25 million and 50 million
17º	Omeprazole	Between 25 million and 50 million
18º	Albendazole	Between 25 million and 50 million
19º	Amlodipine besilate	Between 25 million and 50 million
20º	Clonazepam	Between 25 million and 50 million

Adapted from: ANVISA [1]

Table 2. Occurrence of Pharmaceuticals and Endocrine Disrupting Compounds (P&EDC) in Brazilian waters (raw data).

Compound	N	Range (ng/L)	Matrix	Reference
Acetylsalicylic acid	12	< 0.041 - 15,687.9	RW	[2]
	6	< 0.04 - 5,286.9	DW	[2]
	nr	<LD[a]	RW	[3]
	nr	<LD[a]	DW	[3]
Acyclovir	21	< 0.95 - 220.40	RW	[4]
	20	< 0.95 - 93.08	DW	[4]
Amoxicillin	2	< 0.46 - 8.9	RW	[2]
	72	< 103.8	RW	[3]
	72	< 31.5	DW	[3]
Atenolol	72	< 20.5	RW	[3]
	72	< 14.5	DW	[3]
	nr	< 60,000	RW	[5]
	1	26	RW	[5]
Atorvastatin	72	< 80.8 - 1,020	RW	[3]
	72	< 25.3 - 654	DW	[3]
Betamethasone	72	< 8.0 - 11,960	RW	[3]
	72	< 8.0 - 2,620	DW	[3]
Bezafibrate	9	0.13 - 744.4	RW	[2]
	5	< 2.9 - 1,659.1	DW	[2]
	nr	<LD[a]	RW	[3]
	nr	<LD[a]	DW	[3]
	66	< 71.74 - 1,365.00	RW	[4]
	66	< 71.74 - 436.00	DW	[4]
Bisphenol A	41	< 1.2 - 13,016	RW	[2]
	10	< 1.2 - 2,549.1	DW	[2]
	7	< 58	RW	[6]
	100	< 58	DW	[6]
	3	< 1,200,000	RW	[5]
	10	13 - 43	RW	[5]
	118	< 0.03 - 64,831.00	RW	[4]
	117	< 0.03 - 1,687.00	DW	[4]
Cefalexin	2	< 0.64 - 29	RW	[2]
Cimetidine	2	2.6 - 13,9	RW	[2]
	72	< 24.2	RW	[3]
	72	< 29.6	DW	[3]
Ciprofloxacin	2	< 0.41 - 2.5	RW	[2]
	nr	<LD[a]	RW	[3]
	nr	<LD[a]	DW	[3]
Clarithromycin	72	< 63.5	RW	[3]
	72	< 32.5	DW	[3]
Dexamethasone	79	< 2.86 - 2,159.00	RW	[4]
	78	< 2.86 - 2,271.00	DW	[4]

N: amount of data; RW: raw water; DW: drinking water; nr: not reported in the original paper; a: the limit of detection (LD) of the analytical method (high-performance liquid chromatography - HPLC) was not reported in the original paper.

Table S2 cont. Occurrence of Pharmaceuticals and Endocrine Disrupting Compounds (P&EDC) in Brazilian waters (raw data)

Compound	N	Range (ng/L)	Matrix	Reference
Diclofenac	18	0.14 - 400	RW	[2]
	6	< 4.9 - 330.6	DW	[2]
	nr	<LD[a]	RW	[3]
	nr	<LD[a]	DW	[3]
	54	< 0.28 - 723.20	RW	[4]
	53	< 0.28 - 1,405.00	DW	[4]
Diltiazem	21	< 1.22	RW	[4]
	20	< 1.22	DW	[4]
Enoxacin	72	< 134 - 386	RW	[3]
	72	< 401.6	DW	[3]
Enrofloxacin	72	< 11.8 - 71	RW	[3]
	72	< 5.0 - 219	DW	[3]
17-alpha-Ethinylestradiol	28	0.29 - 4,390	RW	[2]
	6	< 4.61 - 623.0	DW	[2]
	7	< 11	RW	[6]
	100	< 11	DW	[6]
	4	216 - 268	RW	[5]
	118	< 0.39 - 113.90	RW	[4]
	117	< 0.39 - 2.68	DW	[4]
17-beta-Estradiol	28	< 0.6 - 6,806	RW	[2]
	6	< 4.4 - 43.5	DW	[2]
	118	< 0.25 - 75.25	RW	[4]
	117	< 0.25 - 4.30	DW	[4]
Estriol	13	< 0.6 - 67.4	RW	[2]
	4	< 2.1 - 97.4	DW	[2]
	7	< 17	RW	[6]
	100	< 17	DW	[6]
	118	< 0.08 - 9.19	RW	[4]
	117	< 0.08 - 2.10	DW	[4]
Estrone	19	< 0.3 - 78.1	RW	[2]
	6	< 4.7 - 70.1	DW	[2]
	7	< 15	RW	[6]
	100	< 15	DW	[6]
	4	1,930.00 - 2,280.00	RW	[5]
	117	< 0.07 - 279.50	RW	[4]
	117	< 0.07 - 94.80	DW	[4]
Fluconazole	72	< 7.4 - 1,413	RW	[3]
	72	< 8.7 - 750	DW	[3]
Gemfibrozil	12	1.06 - 216.4	RW	[2]
	4	< 1.5 - 98.4	DW	[2]
	72	< 39.6 - 948	RW	[3]
	72	< 85 - 293	DW	[3]
	118	< 0.30 - 2,032.00	RW	[4]
	117	< 0.30 - 2,253.00	DW	[4]

N: amount of data; RW: raw water; DW: drinking water; nr: not reported in the original paper; a: the limit of detection (LD) of the analytical method (high-performance liquid chromatography - HPLC) was not reported in the original paper.

Table S2 cont. Occurrence of Pharmaceuticals and Endocrine Disrupting Compounds (P&EDC) in Brazilian waters (raw data)

Compound	N	Range (ng/L)	Matrix	Reference
Ibuprofen	15	0.02 - 4,155.5	RW	[2]
	6	< 0.66 - 16.9	DW	[2]
	72	< 21.3 - 333	RW	[3]
	72	< 12.3	DW	[3]
	nr	< 125,000	RW	[5]
	118	< 0.28 - 352.60	RW	[4]
	117	< 0.28 - 490.20	DW	[4]
Ketoprofen	72	< 34.7 - 1,020	RW	[3]
	72	< 64.6 - 561	DW	[3]
Levonorgestrel	2	< 19 - 663	RW	[2]
	7	< 1.0	RW	[6]
	100	< 1.0	DW	[6]
Linezolid	21	< 1.75	RW	[4]
	20	< 1.75 - 901.20	DW	[4]
Loratadine	72	< 16.1 - 486	RW	[3]
	72	< 13.6 - 67	DW	[3]
	33	< 1.90 - 44.99	RW	[4]
	32	< 1.90 - 24.34	DW	[4]
Losartan	66	< 1.00 - 926.00	RW	[4]
	66	< 1.00 - 576.40	DW	[4]
Metformin	72	< 1.5 - 203	RW	[3]
	72	< 2.9	DW	[3]
	66	< 1.39 - 176.00	RW	[4]
	66	< 1.39 - 111.20	DW	[4]
Naproxen	10	2.5 - 200	RW	[2]
	nr	< LD[a]	RW	[3]
	nr	< LD[a]	DW	[3]
	54	< 0.2 - 22,408.0	RW	[4]
	53	< 0.2 - 372,632.0	DW	[4]
4-Nonylphenol	20	< 0.5 - 1,918	RW	[2]
	7	< 0.1 - 2,820	DW	[2]
	7	< 6.0	RW	[6]
	100	< 6.0	DW	[6]
	1	< 100,000	RW	[5]
	4	5,620	RW	[5]
	118	< 0.10 - 1,661.00	RW	[4]
	117	< 0.10 - 577.10	DW	[4]
Norfloxacin	2	< 0.4 - 2.2	RW	[2]
	72	< 29.7 - 285	RW	[3]
	72	< 39.3 - 210	DW	[3]

N: amount of data; RW: raw water; DW: drinking water; nr: not reported in the original paper; a: the limit of detection (LD) of the analytical method (high-performance liquid chromatography - HPLC) was not reported in the original paper.

Table S2 cont. Occurrence of Pharmaceuticals and Endocrine Disrupting Compounds (P&EDC) in Brazilian waters (raw data)

Compound	N	Range (ng/L)	Matrix	Reference
4-Octylphenol	16	< 0.1 - 64.6	RW	[2]
	6	< 0.5 - 276.6	DW	[2]
	2	< 100,000	RW	[5]
	4	1,530	RW	[5]
	118	< 0.20 - 835.10	RW	[4]
	117	< 0.20 - 235.00	DW	[4]
Omeprazole	72	< 32.0	RW	[3]
	72	< 17.8	DW	[3]
Paracetamol/ Acetaminophen	14	0.01 - 1,222.6	RW	[2]
	4	< 0.2 - 453.6	DW	[2]
	nr	40,000	RW	[5]
	1	16.0	RW	[5]
	118	< 0.20 - 2,147.00	RW	[4]
	117	< 0.20 - 7.98	DW	[4]
Prednisone	72	< 5.1 - 8,105	RW	[3]
	72	< 4.8 - 6,323	DW	[3]
Promethazine	2	7.0 - 71.9	RW	[2]
	21	< 0.30 - 77.40	RW	[4]
	20	< 0.30 - 30.84	DW	[4]
Propanolol	66	< 8.30 - 271.20	RW	[4]
	66	< 8.30 - 6,837.00	DW	[4]
Ranitidine	2	8.3 - 15.8	RW	[2]
	72	< 66.7	RW	[3]
	72	< 26.7	DW	[3]
Sulfamethoxazole	8	< 0.8 - 1,826.3	RW	[2]
	4	< 1.1 - 2,592.6	DW	[2]
	nr	< LD[a]	RW	[3]
	nr	< LD[a]	DW	[3]
	66	< 2.04 - 275.20	RW	[4]
	66	< 2.04 - 1,052.00	DW	[4]
Tetracycline	2	< 2.5 - 11	RW	[2]
	nr	< LD[a]	RW	[3]
	nr	< LD[a]	DW	[3]
Triclosan	13	< 0.7 - 66	RW	[2]
	7	< 3.0	RW	[6]
	100	< 3.0	DW	[6]
	1	9.0	RW	[5]
Trimethoprim	9	< 0.6 - 1,573.9	RW	[2]
	6	< 0.6 - 4,381.2	DW	[2]
	72	< 17.4	RW	[3]
	72	< 15.1	DW	[3]

N: amount of data; RW: raw water; DW: drinking water; nr: not reported in the original paper; a: the limit of detection (LD) of the analytical method (high-performance liquid chromatography - HPLC) was not reported in the original paper.

Table S3. Occurrence of Pharmaceuticals and Endocrine Disrupting Compounds (P&EDC) in foreign waters (raw data)

Compound	N	Range (ng/L)	Matrix	Country	Reference
Acetylsalicylic acid	31	< LD[a]	RW	Spain	[7]
	nr	150[b]	RW	Netherlands	[8]
	nr	<LD[c]	DW	Netherlands	[8]
	nr	1,130	RW	South Africa	[9]
Acyclovir	9	< 10	RW	Germany	[10]
	5	< 10	DW	Germany	[10]
Amoxicillin	31	< LD[a]	RW	Spain	[7]
	21	< LD[d]	DW	Italy	[11]
Atenolol	19	< 0.25 - 36	RW	USA	[12]
	19	< 0.25 - 18	DW	USA	[12]
	36	< 5 - 900	RW	Spain	[13]
	36	< 5 - 715	DW	Spain	[13]
	6	< 3.56 - 8.55	RW	Italy	[14]
	6	< 3.56	DW	Italy	[14]
	31	< LD[a] - 178	RW	Spain	[7]
	21	< LD[d]	DW	Italy	[11]
	6	< 1.26	RW	China	[15]
	3	< 1.26	RW	Sweden	[15]
	3	10.96	RW	Spain	[15]
	3	3.64	RW	Czech Republic	[15]
	3	< 1.26	RW	Netherlands	[15]
	3	1.02	RW	Switzerland	[15]
	3	< 1.26	RW	Germany	[15]
	6	0.92 - 7.16	RW	Italy	[15]
	3	< 1.26	RW	Vietnam	[15]
	3	< 1.26	RW	Belgium	[15]
	3	4.82	RW	Japan	[15]
	nr	< 91[b]	RW	Netherlands	[8]
	19	< LD[e] - 941.1	RW	Bangladesh	[16]
	1	35	RW	USA	[17]
	nr	3.6	RW	Taiwan	[9]
	nr	< 0.4 - 19.5	DW	USA	[9]
Atorvastatin	19	< 0.50 - 1.4	RW	USA	[12]
	18	< 0.5	DW	USA	[12]
	31	< LD[a]	RW	Spain	[7]
	21	< LD[d]	DW	Italy	[11]
	1	< 0.7	RW	USA	[17]
	nr	< 530[b]	RW	Netherlands	[8]
	nr	< LD[c]	DW	Netherlands	[8]
	nr	< LD[f] - 0.6	RW	China	[9]
	nr	<0.1 - 1.5	RW	Serbia	[9]

N: amount of data; RW: raw water; DW: drinking water; nr: not reported in the original paper; Data were reported as <LD or <LQ when the limit of detection (LD) and/or the limit of quantification (LQ) of the analytical method were not informed in the original paper. a: samples analyzed by solid-phase extraction (SPE) followed by ultra-performance liquid chromatography-triple quadrupole mass spectrometry (UPLC-QqQ-MS/MS); b: maximum concentration; c: analytical method not informed; d: samples analyzed by liquid chromatography coupled with mass spectrometry (LC-MS); e: samples analyzed by liquid chromatography coupled with high-resolution mass spectrometry (LC-HRMS); f: samples analyzed by SPE followed by LC with tandem MS (SPE-HPLC-MS/MS).

Table S3 cont. Occurrence of Pharmaceuticals and Endocrine Disrupting Compounds (P&EDC) in foreign waters (raw data)

Compound	N	Range (ng/L)	Matrix	Country	Reference
Betamethasone	17	0.29 - 7.2	RW	China	[18]
	11	< 0.02 - 1.0	DW	Germany	[19]
Bezafibrate	31	< LD[a]	RW	Spain	[7]
	21	< LD[b]	DW	Italy	[11]
	6	< 1.8	RW	China	[15]
	3	< 1.8	RW	Sweden	[15]
	3	6.65	RW	Spain	[15]
	3	< 1.8	RW	Czech Republic	[15]
	3	< 1.8	RW	Netherlands	[15]
	3	< 1.8	RW	Switzerland	[15]
	3	< 1.8	RW	Germany	[15]
	6	< 1.8	RW	Italy	[15]
	3	< 1.8	RW	Vietnam	[15]
	3	< 1.8	RW	Belgium	[15]
	3	5.44	RW	Japan	[15]
	nr	< 80[c]	RW	Netherlands	[8]
	nr	< LD[d]	DW	Netherlands	[8]
	nr	< LD[e] - 31.4	RW	China	[9]
	nr	< 0.01 - 0.16	DW	China	[9]
	nr	0.1 - 256.7	RW	Taiwan	[9]
Bisphenol A	19	< 5 - 11	RW	USA	[12]
	18	< 5 - 10	DW	USA	[12]
	12	nr	RW	USA	[20]
	4	< 1,000	DW	USA	[20]
	6	< 0.99	RW	Italy	[14]
	6	< 0.99 - 6.27	DW	Italy	[14]
	31	< LD[f]	RW	Spain	[7]
	21	< 1.06 - 683	DW	Italy	[11]
	nr	< 10.3 - 763	RW	USA	[9]
	nr	< 10.3 - 421	DW	USA	[9]
Cefalexin	nr	< 1.1 - 283	RW	Serbia	[9]
Cimetidine	1	< 1.8	RW	USA	[17]

N: amount of data; RW: raw water; DW: drinking water; nr: not reported in the original paper; Data were reported as <LD or <LQ when the limit of detection (LD) and/or the limit of quantification (LQ) of the analytical method were not informed in the original paper. a: samples analyzed by solid-phase extraction (SPE) followed by ultra-performance liquid chromatography-triple quadrupole mass spectrometry (UPLC-QqQ-MS/MS); b: samples analyzed by liquid chromatography coupled with mass spectrometry (LC-MS); c: maximum concentration; d: analytical method not informed; e: samples analyzed by SPE followed by LC with tandem MS (SPE-HPLC-MS/MS); f: samples analyzed by stir bar sorptive extraction (SBSE) followed by gas chromatography coupled to tandem mass spectrometry (GC-MS/MS)

Table S3 cont. Occurrence of Pharmaceuticals and Endocrine Disrupting Compounds (P&EDC) in foreign waters (raw data)

Compound	N	Range (ng/L)	Matrix	Country	Reference
Ciprofloxacin	12	nr	RW	USA	[20]
	4	< 20	DW	USA	[20]
	31	< LD[a]	RW	Spain	[7]
	21	< LD[b]	DW	Italy	[11]
	19	< LD[c] - 1,407	RW	Bangladesh	[16]
	4	61.5 - 635.4	RW	India	[21]
	1	< LD[c]	RW	Philippines	[21]
	4	< LD[c]	RW	USA	[21]
	4	< LD[c]	RW	Switzerland	[21]
	4	< LD[c] - 9.4	RW	Sweden	[21]
	nr	1.6	RW	Taiwan	[9]
	nr	< 5.5 - 28.2	RW	Serbia	[9]
	nr	1.5 - 2.4	RW	Sweden	[9]
	nr	88.7	RW	Portugal	[9]
Clarithromycin	31	< LD[a]	RW	Spain	[7]
	21	< LD[b]	DW	Italy	[11]
	6	< 1.22	RW	China	[15]
	3	< 1.22	RW	Sweden	[15]
	3	24.06	RW	Spain	[15]
	3	27.75	RW	Czech Republic	[15]
	3	< 1.22	RW	Netherlands	[15]
	3	1.73	RW	Switzerland	[15]
	3	< 1.22	RW	Germany	[15]
	6	3.13 - 3.87	RW	Italy	[15]
	3	< 1.22	RW	Vietnam	[15]
	3	0.66	RW	Belgium	[15]
	3	18.48	RW	Japan	[15]
	nr	< LD[d]	RW	Netherlands	[8]
	nr	< LD[d]	DW	Netherlands	[8]
	19	< LD[c] - 205	RW	Bangladesh	[16]
	4	< LD[c] - 9831.5	RW	India	[21]
	1	23.3	RW	Philippines	[21]
	4	< LD[c] - 1.8	RW	USA	[21]
	4	< LD[c] - 13.5	RW	Switzerland	[21]
	4	< LD[c] - 12.0	RW	Sweden	[21]
	nr	6.9 - 12.5	RW	Taiwan	[9]
	nr	123	RW	China	[9]
	nr	26.8	RW	Portugal	[9]
	nr	< 0.6 - 616	RW	Serbia	[9]

N: amount of data; RW: raw water; DW: drinking water; nr: not reported in the original paper; Data were reported as <LD or <LQ when the limit of detection (LD) and/or the limit of quantification (LQ) of the analytical method were not informed in the original paper. a: samples analyzed by solid-phase extraction (SPE) followed by ultra-performance liquid chromatography-triple quadrupole mass spectrometry (UPLC-QqQ-MS/MS); b: samples analyzed by liquid chromatography coupled with mass spectrometry (LC-MS); c: samples analyzed by liquid chromatography coupled to triple quadrupole mass spectrometry (LC-MS/MS); d: analytical method not informed.

Table S3 cont. Occurrence of Pharmaceuticals and Endocrine Disrupting Compounds (P&EDC) in foreign waters (raw data)

Compound	N	Range (ng/L)	Matrix	Country	Reference
Dexamethasone	nr	< LD[a]	RW	Netherlands	[8]
	nr	< LD[a]	DW	Netherlands	[8]
	17	0.51 - 9.7	RW	China	[18]
	11	< 0.02 - < 0.05	DW	Germany	[19]
	nr	0.0004	RW	Sweden	[9]
Diclofenac	19	< 0.25 - 1.2	RW	USA	[12]
	18	< 0.25	DW	USA	[12]
	6	< 0.51 - 15.91	RW	Italy	[14]
	6	< 0.51	DW	Italy	[14]
	1	< 5.36	RW	India	[22]
	31	< LD[b] - 83	RW	Spain	[7]
	21	< LD[c]	DW	Italy	[11]
	6	< 10.99	RW	China	[15]
	3	< 10.99	RW	Sweden	[15]
	3	36.77	RW	Spain	[15]
	3	28.26	RW	Czech Republic	[15]
	3	< 10.99	RW	Netherlands	[15]
	3	< 10.99	RW	Switzerland	[15]
	3	< 10.99	RW	Germany	[15]
	6	< 10.99	RW	Italy	[15]
	3	< 10.99	RW	Vietnam	[15]
	3	< 10.99	RW	Belgium	[15]
	3	< 10.99	RW	Japan	[15]
	nr	< 180[b]	RW	Netherlands	[8]
	nr	< LD[a]	DW	Netherlands	[8]
	19	< LD[d] - 57	RW	Bangladesh	[16]
	nr	< LD[e] - 1.5	RW	China	[9]
	nr	< 0.02 - 2.37	DW	China	[9]
	nr	2.1 - 33.2	RW	Taiwan	[9]
	nr	38	RW	Portugal	[9]
	nr	< 4.1 - 324	RW	Serbia	[9]
	nr	1,010 - 10,200	RW	South Africa	[9]
	nr	1.7 - 3.6	RW	Sweden	[9]
	nr	0.41 - 0.31	RW	Vietnam	[9]
Diltiazem	nr	5.6	RW	China	[9]
	nr	< 0.5 - 6.9	RW	Serbia	[9]
Enoxacin	nr	< 2,250[f]	RW	India	[23]
	nr	< 200[f]	RW	China	[23]

N: amount of data; RW: raw water; DW: drinking water; nr: not reported in the original paper; Data were reported as <LD or <LQ when the limit of detection (LD) and/or the limit of quantification (LQ) of the analytical method were not informed in the original paper. a: analytical method not informed; b: samples analyzed by solid-phase extraction (SPE) followed by ultra-performance liquid chromatography-triple quadrupole mass spectrometry (UPLC-QqQ-MS/MS); c: samples analyzed by liquid chromatography coupled with mass spectrometry (LC-MS); d: samples analyzed by liquid chromatography coupled to triple quadrupole mass spectrometry (LC-MS/MS); e: samples analyzed by SPE followed by LC with tandem MS (SPE-HPLC-MS/MS); f: maximum concentration.

Table S3 cont. Occurrence of Pharmaceuticals and Endocrine Disrupting Compounds (P&EDC) in foreign waters (raw data)

Compound	N	Range (ng/L)	Matrix	Country	Reference
Enrofloxacin	31	< LD[a]	RW	Spain	[7]
	4	< LD[b] - 142.3	RW	India	[21]
	1	< LD[b]	RW	Philippines	[21]
	4	< LD[b]	RW	USA	[21]
	4	< LD[b]	RW	Switzerland	[21]
	4	< LD[b]	RW	Sweden	[21]
	nr	< 0.51 - 6.6	RW	China	[9]
Estradiol	6	< 0.81 - 4.04	RW	Italy	[14]
	6	< 0.81	DW	Italy	[14]
	31	< LD[a]	RW	Spain	[7]
	21	< LD[c]	DW	Italy	[11]
	nr	0.0011–0.003	RW	Sweden	[9]
Ethinylestradiol	36	< 0.2 - 3.4	RW	Spain	[13]
	36	< 0.2	DW	Spain	[13]
	6	< 2.66	RW	Italy	[14]
	6	< 2.66	DW	Italy	[14]
	31	< LD[c]	RW	Spain	[7]
	21	< LD[d]	DW	Italy	[11]
	nr	0.001–0.0016	RW	Sweden	[9]
Estriol	36	< 4.7 - 72	RW	Spain	[13]
	36	< 4.7	DW	Spain	[13]
Estrone	19	< 0.20 - 0.94	RW	USA	[12]
	18	< 0.20	DW	USA	[12]
	36	< 0.2 - 1.0	RW	Spain	[13]
	36	< 0.2	DW	Spain	[13]
	6	< 0.92	RW	Italy	[14]
	6	< 0.92	DW	Italy	[14]
	31	< LD[c]	RW	Spain	[7]
	21	< LD[d]	DW	Italy	[11]
	nr	< 130[e]	RW	Netherlands	[8]
	nr	< LD[f]	DW	Netherlands	[8]

N: amount of data; RW: raw water; DW: drinking water; nr: not reported in the original paper; Data were reported as <LD or <LQ when the limit of detection (LD) and/or the limit of quantification (LQ) of the analytical method were not informed in the original paper. a: samples analyzed by solid-phase extraction (SPE) followed by ultra-performance liquid chromatography-triple quadrupole mass spectrometry (UPLC-QqQ-MS/MS); b: samples analyzed by liquid chromatography coupled to triple quadrupole mass spectrometry (LC-MS/MS); c: samples analyzed by stir bar sorptive extraction (SBSE) followed by gas chromatography coupled to tandem mass spectrometry (GC-MS/MS); d: samples analyzed by liquid chromatography coupled with mass spectrometry (LC-MS); e: maximum concentration; f: analytical method not informed.

Table S3 cont. Occurrence of Pharmaceuticals and Endocrine Disrupting Compounds (P&EDC) in foreign waters (raw data)

Compound	N	Range (ng/L)	Matrix	Country	Reference
Fluconazole	6	11.93 - 17.39	RW	China	[15]
	3	< 0.30	RW	Sweden	[15]
	3	204.48	RW	Spain	[15]
	3	< 0.30	RW	Czech Republic	[15]
	3	< 0.30	RW	Netherlands	[15]
	3	< 0.30	RW	Switzerland	[15]
	3	< 0.30	RW	Germany	[15]
	6	< 0.30 - 3.25	RW	Italy	[15]
	3	< 0.30	RW	Vietnam	[15]
	3	20.86	RW	Belgium	[15]
	3	< 0.30	RW	Japan	[15]
	19	< LD[a] - 898.8	RW	Bangladesh	[16]
Gemfibrozil	19	< 0.25 - 24	RW	USA	[12]
	18	< 0.25 - 2.1	DW	USA	[20]
	12	nr	RW	USA	[20]
	4	< 15	DW	USA	[20]
	31	< LD[b]	RW	Spain	[7]
	21	< LD[c]	DW	Italy	[11]
	1	< 12	RW	USA	[17]
	nr	< 210[d]	RW	Netherlands	[8]
	nr	< 300[d]	DW	Netherlands	[8]
	nr	0.1 - 172.3	RW	Taiwan	[9]
	nr	< 0.01 - 2.3	RW	China	[9]
	nr	< 0.05	RW	Vietnam	[9]

N: amount of data; RW: raw water; DW: drinking water; nr: not reported in the original paper; Data were reported as <LD or <LQ when the limit of detection (LD) and/or the limit of quantification (LQ) of the analytical method were not informed in the original paper. a: samples analyzed by liquid chromatography coupled with high-resolution mass spectrometry (LC-HRMS); b: samples analyzed by solid-phase extraction (SPE) followed by ultra-performance liquid chromatography-triple quadrupole mass spectrometry (UPLC-QqQ-MS/MS); c: samples analyzed by liquid chromatography coupled with mass spectrometry (LC-MS); d: maximum concentration.

Table S3 cont. Occurrence of Pharmaceuticals and Endocrine Disrupting Compounds (P&EDC) in foreign waters (raw data)

Compound	N	Range (ng/L)	Matrix	Country	Reference
Ibuprofen	12	nr	RW	USA	[20]
	4	< 18	DW	USA	[20]
	6	< 1.96 - 15.57	RW	Italy	[14]
	6	< 1.96	DW	Italy	[14]
	31	< LD[a] - 89	RW	Spain	[7]
	21	< LD[b]	DW	Italy	[11]
	1	< 15	RW	USA	[17]
	nr	< 104[c]	RW	Netherlands	[8]
	nr	< LD[d]	DW	Netherlands	[8]
	nr	< LD[e] - 242.0	RW	China	[9]
	nr	< 1.8 - 17.17	DW	China	[9]
	nr	< 3.2 - 92	RW	Serbia	[9]
	nr	7.0 - 836.7	RW	Taiwan	[9]
	nr	1,317	RW	Portugal	[9]
	nr	< 0.85 - 1.16	DW	USA	[9]
	nr	< 3.2 - 346	RW	Serbia	[9]
	nr	5.0 - 62	RW	Colombia	[9]
	nr	524 - 17,600	RW	South Africa	[9]
	nr	2.2	RW	Sweden	[9]
Ketoprofen	31	< LD[a] - 1,481	RW	Spain	[7]
	21	< LD[b]	DW	Italy	[11]
	6	< 0.89	RW	China	[15]
	3	< 0.89	RW	Sweden	[15]
	3	15.34	RW	Spain	[15]
	3	< 0.89	RW	Czech Republic	[15]
	3	< 0.89	RW	Netherlands	[15]
	3	< 0.89	RW	Switzerland	[15]
	3	< 0.89	RW	Germany	[15]
	6	< 0.89	RW	Italy	[15]
	3	< 0.89	RW	Vietnam	[15]
	3	< 0.89	RW	Belgium	[15]
	3	< 0.89	RW	Japan	[15]
	nr	< 20[c]	RW	Netherlands	[8]
	nr	< 40[c]	DW	Netherlands	[8]
	nr	< 9.0 - 45	RW	Serbia	[9]
	nr	< 9.0 - 16	DW	Serbia	[9]
	nr	1.4 - 54.5	RW	China	[9]
	nr	75.3	RW	Portugal	[9]
	nr	443 - 9,220	RW	South Africa	[9]
	nr	0.29 - 1.3	RW	Sweden	[9]
	nr	< 0.04 - 0.45	RW	Vietnam	[9]

N: amount of data; RW: raw water; DW: drinking water nr: not reported in the original paper; Data were reported as <LD or <LQ when the limit of detection (LD) and/or the limit of quantification (LQ) of the analytical method were not informed in the original paper. a: samples analyzed by solid-phase extraction (SPE) followed by ultra-performance liquid chromatography-triple quadrupole mass spectrometry (UPLC-QqQ-MS/MS); b: samples analyzed by liquid chromatography coupled with mass spectrometry (LC-MS); c: maximum concentration; d: analytical method not informed; e: samples analyzed by SPE followed by LC with tandem MS (SPE-HPLC-MS/MS).

Table S3 cont. Occurrence of Pharmaceuticals and Endocrine Disrupting Compounds (P&EDC) in foreign waters (raw data)

Compound	N	Range (ng/L)	Matrix	Country	Reference
Levonorgestrel	6	< 0.42	RW	China	[15]
	3	< 0.42	RW	Sweden	[15]
	3	< 0.42	RW	Spain	[15]
	3	< 0.42	RW	Czech Republic	[15]
	3	< 0.42	RW	Netherlands	[15]
	3	< 0.42	RW	Switzerland	[15]
	3	< 0.42	RW	Germany	[15]
	6	< 0.42	RW	Italy	[15]
	3	< 0.42	RW	Vietnam	[15]
	3	< 0.42	RW	Belgium	[15]
	3	< 0.42	RW	Japan	[15]
	11	< 0.05 - 0.7	DW	Germany	[19]
Linezolid	19	< LD[a] - 87.6	RW	Bangladesh	[16]
Loratadine	6	< 0.03	RW	China	[15]
	3	< 0.03	RW	Sweden	[15]
	3	0.40	RW	Spain	[15]
	3	< 0.03	RW	Czech Republic	[15]
	3	< 0.03	RW	Netherlands	[15]
	3	< 0.03	RW	Switzerland	[15]
	3	< 0.03	RW	Germany	[15]
	6	< 0.03	RW	Italy	[15]
	3	0.04	RW	Vietnam	[15]
	3	< 0.03	RW	Belgium	[15]
	3	< 0.03	RW	Japan	[15]
	nr	< 0.05 - 0.6	RW	China	[9]

N: amount of data; RW: raw water; DW: drinking water; nr: not reported in the original paper; Data were reported as <LD or <LQ when the limit of detection (LD) and/or the limit of quantification (LQ) of the analytical method were not informed in the original paper. a: samples analyzed by liquid chromatography coupled with high-resolution mass spectrometry (LC-HRMS).

Table S3 cont. Occurrence of Pharmaceuticals and Endocrine Disrupting Compounds (P&EDC) in foreign waters (raw data)

Compound	N	Range (ng/L)	Matrix	Country	Reference
Losartan	36	< 14 - 620	RW	Spain	[13]
	36	< 14 - 150	DW	Spain	[13]
	6	< 0.11 - 5.23	RW	China	[15]
	3	< 0.11	RW	Sweden	[15]
	3	148.28	RW	Spain	[15]
	3	9.21	RW	Czech Republic	[15]
	3	< 0.11	RW	Netherlands	[15]
	3	3.41	RW	Switzerland	[15]
	3	< 0.11	RW	Germany	[15]
	6	1.06 - 4.99	RW	Italy	[15]
	3	4.06	RW	Vietnam	[15]
	3	3.71	RW	Belgium	[15]
	3	9.98	RW	Japan	[15]
	nr	< 120[a]	RW	Netherlands	[8]
	nr	< LD[b]	DW	Netherlands	[8]
	19	< LD[c] - 92.6	RW	Bangladesh	[16]
	nr	< 0.05 - 1.2	RW	China	[9]
	nr	< 3.5 - 154	RW	Serbia	[9]
Metformin	nr	< 3,200[a]	RW	Netherlands	[8]
	nr	< LD[b]	DW	Netherlands	[8]
	nr	8.4	RW	Sweden	[9]
Naproxen	19	< 0.5 - 32	RW	USA	[12]
	19	< 0.5	DW	USA	[12]
	31	< LD[d]	RW	Spain	[7]
	21	< LD[e]	DW	Italy	[11]
	nr[a]	< 40[a]	RW	Netherlands	[8]
	nr	< LD[b]	DW	Netherlands	[8]
	nr	3.5	RW	China	[9]
	nr	< 0.1 - 3.12	DW	China	[9]
	nr	< 1.3 - 74.2	RW	Serbia	[9]
	nr	128.0	RW	Taiwan	[9]
	nr	260	RW	Portugal	[9]
	nr	< 1.0 - 10.85	RW	Singapore	[9]
	nr	59,300	RW	South Africa	[9]
	nr	0.220	RW	Sweden	[9]
4-Nonylphenol	19	< 80 - 130	RW	USA	[12]
	6	< 2.05 - 53.62	RW	Italy	[14]
	6	< 2.05	DW	Italy	[14]
	31	< LD[f]	RW	Spain	[7]
	21	< 13.2 - 16	DW	Italy	[11]

N: amount of data; RW: raw water; DW: drinking water; nr: not reported in the original paper; Data were reported as <LD or <LQ when the limit of detection (LD) and/or the limit of quantification (LQ) of the analytical method were not informed in the original paper. a: maximum concentration; b: analytical method not reported; c: samples analyzed by liquid chromatography coupled with high-resolution mass spectrometry (LC-HRMS); d: samples analyzed by solid-phase extraction (SPE) followed by ultra-performance liquid chromatography-triple quadrupole mass spectrometry (UPLC-QqQ-MS/MS); e: samples analyzed by liquid chromatography coupled with mass spectrometry (LC-MS); f: samples analyzed by stir bar sorptive extraction (SBSE) followed by gas chromatography coupled to tandem mass spectrometry (GC-MS/MS).

Table S3 cont. Occurrence of Pharmaceuticals and Endocrine Disrupting Compounds (P&EDC) in foreign waters (raw data)

Compound	N	Range (ng/L)	Matrix	Country	Reference
Norfloxacin	12	nr	RW	USA	[20]
	4	< 20	DW	USA	[20]
	31	< LD[a]	RW	Spain	[7]
	6	< 6.64	RW	China	[15]
	3	< 6.64	RW	Sweden	[15]
	3	< 6.64	RW	Spain	[15]
	3	< 6.64	RW	Czech Republic	[15]
	3	< 6.64	RW	Netherlands	[15]
	3	< 6.64	RW	Switzerland	[15]
	3	< 6.64	RW	Germany	[15]
	6	< 6.64	RW	Italy	[15]
	3	< 6.64	RW	Vietnam	[15]
	3	< 6.64	RW	Belgium	[15]
	3	< 6.64	RW	Japan	[15]
	nr	< 9.0[b]	RW	Netherlands	[8]
	nr	< LD[c]	DW	Netherlands	[8]
	4	< LD[d] - 261	RW	India	[21]
	1	< LD[d]	RW	Philippines	[21]
	4	< LD[d]	RW	USA	[21]
	4	< LD[d]	RW	Switzerland	[21]
	4	< LD[d]	RW	Sweden	[21]
	nr	< LD[e] - 92.4	RW	China	[9]
	nr	2.8 - 9.3	RW	Taiwan	[9]
4-Octylphenol	6	< 0.66	RW	Italy	[14]
	6	< 0.66	DW	Italy	[14]
	31	< LD[f]	RW	Spain	[7]
	21	< LD[g]	DW	Italy	[11]
	nr	< LD[c]	RW	Netherlands	[8]
	nr	< LD[c]	DW	Netherlands	[8]

N: amount of data; RW: raw water; DW: drinking water; nr: not reported in the original paper; Data were reported as <LD or <LQ when the limit of detection (LD) and/or the limit of quantification (LQ) of the analytical method were not informed in the original paper. a: samples analyzed by solid-phase extraction (SPE) followed by ultra-performance liquid chromatography-triple quadrupole mass spectrometry (UPLC-QqQ-MS/MS); b: maximum concentration; c: analytical method not informed; d: samples analyzed by liquid chromatography coupled to triple quadrupole mass spectrometry (LC-MS/MS); e: samples analyzed by SPE followed by LC with tandem MS (SPE-HPLC-MS/MS); f: samples analyzed by stir bar sorptive extraction (SBSE) followed by gas chromatography coupled to tandem mass spectrometry (GC-MS/MS); g: samples analyzed by liquid chromatography coupled with mass spectrometry (LC-MS).

Table S3 cont. Occurrence of Pharmaceuticals and Endocrine Disrupting Compounds (P&EDC) in foreign waters (raw data)

Compound	N	Range (ng/L)	Matrix	Country	Reference
Omeprazole	6	< 2.96	RW	China	[15]
	3	< 2.96	RW	Sweden	[15]
	3	< 2.96	RW	Spain	[15]
	3	< 2.96	RW	Czech Republic	[15]
	3	< 2.96	RW	Netherlands	[15]
	3	< 2.96	RW	Switzerland	[15]
	3	< 2.96	RW	Germany	[15]
	6	< 2.96	RW	Italy	[15]
	3	< 2.96	RW	Vietnam	[15]
	3	< 2.96	RW	Belgium	[15]
	3	< 2.96	RW	Japan	[15]
	21	< LD[a]	DW	Italy	[11]
	nr	1.03	RW	Sweden	[9]
Paracetamol/ Acetaminophen	31	< LD[b] - 200	RW	Spain	[7]
	21	< LD[a]	DW	Italy	[11]
	6	< 1.83	RW	China	[15]
	3	< 1.83	RW	Sweden	[15]
	3	< 1.83	RW	Spain	[15]
	3	< 1.83	RW	Czech Republic	[15]
	3	< 1.83	RW	Netherlands	[15]
	3	< 1.83	RW	Switzerland	[15]
	3	< 1.83	RW	Germany	[15]
	6	< 1.83 - 17.01	RW	Italy	[15]
	3	< 1.83	RW	Vietnam	[15]
	3	< 1.83	RW	Belgium	[15]
	3	< 1.83	RW	Japan	[15]
	nr	< 1,401[c]	RW	Netherlands	[8]
	nr	< 7.0[c]	DW	Netherlands	[8]
	1	< 6.6	RW	USA	[17]
	19	< LD[d] - 43,518	RW	Bangladesh	[16]
	nr	0.65	RW	Sweden	[9]
	nr	< 4.0 - 159	RW	Singapore	[9]
	nr	527	RW	Portugal	[9]
	nr	< 0.1 - 445.6	RW	China	[9]
	nr	0.9 - 1,036	RW	Taiwan	[9]
Prednisone	nr	< LD[e]	RW	Netherlands	[8]
	nr	< LD[e]	DW	Netherlands	[8]
	17	< 0.02 - 1.3	RW	China	[18]
	11	< 0.03 - 0.05	DW	Germany	[19]
Promethazine	1	< 0.2	RW	USA	[17]

N: amount of data; RW: raw water; DW: drinking water; nr: not reported in the original paper; Data were reported as <LD or <LQ when the limit of detection (LD) and/or the limit of quantification (LQ) of the analytical method were not informed in the original paper. a: samples analyzed by liquid chromatography coupled with mass spectrometry (LC-MS); b: samples analyzed by solid-phase extraction (SPE) followed by ultra-performance liquid chromatography-triple quadrupole mass spectrometry (UPLC-QqQ-MS/MS); c: maximum concentration; d: samples analyzed by liquid chromatography coupled to triple quadrupole mass spectrometry (LC-MS/MS); e: analytical method not informed.

Table S3 cont. Occurrence of Pharmaceuticals and Endocrine Disrupting Compounds (P&EDC) in foreign waters (raw data)

Compound	N	Range (ng/L)	Matrix	Country	Reference
Propanolol	54	< 1.1 - 270	RW	Spain	[13]
	26	< 1.1 - 130	DW	Spain	[13]
	31	< LD[a] - 537	RW	Spain	[7]
Ranitidine	31	< LD[a] - 498	RW	Spain	[7]
	nr	< 0.1 - 54.4	RW	Serbia	[9]
	1	< 5.0	RW	USA	[17]
Sulfamethoxazole	19	< 0.25 - 110	RW	USA	[12]
	18	< 0.25	DW	USA	[12]
	12	nr	RW	USA	[20]
	4	< 50	DW	USA	[20]
	31	< LD[a] - 128	RW	Spain	[7]
	21	< LD[b]	DW	Italy	[11]
	6	< 0.69 - 3.93	RW	China	[15]
	3	< 0.69	RW	Sweden	[15]
	3	53.18	RW	Spain	[15]
	3	13.76	RW	Czech Republic	[15]
	3	< 0.69	RW	Netherlands	[15]
	3	6.85	RW	Switzerland	[15]
	3	< 0.69	RW	Germany	[15]
	6	2.38 - 3.16	RW	Italy	[15]
	3	5.77	RW	Vietnam	[15]
	3	6.88	RW	Belgium	[15]
	3	16.69	RW	Japan	[15]
	nr	< 76[c]	RW	Netherlands	[8]
	nr	< LD[d]	DW	Netherlands	[8]
	19	< LD[e] - 728	RW	Bangladesh	[16]
	4	< LD[f]	RW	India	[21]
	1	69.5	RW	Philippines	[21]
	4	< LD[f] - 21.5	RW	USA	[21]
	4	< LD[f] - 52.8	RW	Switzerland	[21]
	4	< LD[f] - 4.0	RW	Sweden	[21]
	1	140	RW	USA	[17]
	nr	< 0.11 - 115.3	RW	China	[9]
	nr	< 0.02 - 1.81	DW	China	[9]
	nr	0.1 - 1,820	RW	Taiwan	[9]
	nr	< 0.32 - 3.21	RW	USA	[9]
	nr	< 0.03 - 7.24	RW	Bangladesh	[9]
	nr	43	RW	Portugal	[9]
	nr	0.075 - 0.14	RW	Sweden	[9]
Tetracycline	21	< LD[b]	DW	Italy	[11]

N: amount of data; RW: raw water; DW: drinking water; nr: not reported in the original paper; Data were reported as <LD or <LQ when the limit of detection (LD) and/or the limit of quantification (LQ) of the analytical method were not informed in the original paper. a: samples analyzed by solid-phase extraction (SPE) followed by ultra-performance liquid chromatography-triple quadrupole mass spectrometry (UPLC-QqQ-MS/MS); b: samples analyzed by liquid chromatography coupled with mass spectrometry (LC-MS); c: maximum concentration; d: analytical method not informed; e: samples analyzed by liquid chromatography coupled to triple quadrupole mass spectrometry (LC-MS/MS); f: samples analyzed by liquid chromatography coupled to triple quadrupole mass spectrometry (LC-MS/MS)

Table S3 cont. Occurrence of Pharmaceuticals and Endocrine Disrupting Compounds (P&EDC) in foreign waters (raw data)

Compound	N	Range (ng/L)	Matrix	Country	Reference
Triclosan	19	< 1.0 - 6.4	RW	USA	[12]
	18	< 1.0 - 1.2	DW	USA	[12]
	12	nr	RW	USA	[20]
	4	< 1,000	DW	USA	[20]
	31	< LD[a] - 102	RW	Spain	[7]
	nr	< 1.17 - 4.76	RW	USA	[9]
	nr	< 1.17 - 1.93	DW	USA	[9]
	nr	1.8 - 64.5	RW	China	[9]
Trimethoprim	19	< 0.25 - 11	RW	USA	[12]
	18	< 0.25	DW	USA	[12]
	12	nr	RW	USA	[20]
	4	< 14	DW	USA	[20]
	31	< LQ[a] - 176	RW	Spain	[7]
	nr	< 43[b]	RW	Netherlands	[8]
	nr	< LD[c]	DW	Netherlands	[8]
	19	< LD[d] - 153	RW	Bangladesh	[16]
	nr	0.1 - 17.8	RW	Taiwan	[9]
	nr	< 0.08 - 17.20	RW	Bangladesh	[9]
	nr	0.4 - 156	RW	China	[9]
	nr	< 0.6 - 8.1	RW	Serbia	[9]
	nr	0.33	RW	Sweden	[9]
	nr	< 0.37 - 0.68	RW	USA	[9]

N: amount of data; RW: raw water; DW: drinking water; nr: not reported in the original paper; Data were reported as <LD or <LQ when the limit of detection (LD) and/or the limit of quantification (LQ) of the analytical method were not informed in the original paper. a: samples analyzed by solid-phase extraction (SPE) followed by ultra-performance liquid chromatography-triple quadrupole mass spectrometry (UPLC-QqQ-MS/MS); b: maximum concentration; c: analytical method not informed; d: samples analyzed by liquid chromatography coupled to triple quadrupole mass spectrometry (LC-MS/MS).

Table S4. Toxicological studies and guideline values (GV) for selected pharmaceutical and endocrine disrupting compounds (P&EDC).

CEC	Reference	Type of study	Critical effect	Experimental dose (μg/kg bw.d)	UF	ADI (μg/kg bw.d)	AF[1]	GV[1] (μg/L)
Acyclovir (antiviral)	[24]	Lowest daily therapeutic dose (800 mg/d).	Therapeutic effect.	13,300 (LDTD)	1,000	13.3	1.0	400
Acetylsalicylic acid (nonsteroidal anti-inflammatory)	[25]	Epidemiological.	Side effects.	143 (LOAEL)	20	7.2	0.1	22
Albendazole (antiparasitic)	[24]	Lowest daily therapeutic dose (400 mg/d).	Therapeutic effect.	6,666 (LDTD)	1,000	6.7	0.1	20
Amoxicillin (antibiotic)	[26]	Study in humans.	Allergic reactions to penicillins (30 μg/d), and also applicable to amoxicillin.	0.5 (NOAEL)	1	0.5	0.1	1.5
Atenolol (antihypertensive)	[12]	Toxicological study in humans which assessed the gestation period.	Developmental.	800 (LOAEL)	300	2.7	1.0	80
	[25]	Lower daily therapeutic dose (50 mg/d).	Therapeutic effect.	830 (LDTD)	1,000	0.8	1.0	25

ADI: Acceptable daily intake; AF: allocation factor of the ADI for the exposure via drinking water consumption; UF: uncertainty factor; NOAEL: no-observed-adverse-effect levels: highest level of exposure for which no adverse effects are observed; LOAEL: lowest-observed-adverse-effect levels: lowest exposure level for which adverse effects are observed; LDTD: Lowest daily therapeutic dose of a drug; BMDL10: lower limit of critical effect of the benchmark dose (related to the lower limit at a 95% confidence interval for the level of exposure that increases by 10% the risk below the least adverse effect).

[1] The values do not correspond to the original references since in this work they were calculated considering specific premises and factors adjusted to the Brazilian reality (see item 2.2 - Estimation of guideline values for P&EDC in drinking waters).

Table S4 cont. Toxicological studies and guideline values (GV) for selected pharmaceutical and endocrine disrupting compounds (P&EDC).

CEC	Reference	Type of study	Critical effect	Experimental dose (µg/kg bw.d)	UF	ADI (µg/kg bw.d)	AF[1]	GV[1] (µg/L)
Atorvastatin (antilipemic)	[12]	Toxicological study in rats which assessed the gestation and postnatal period.	Developmental.	20,000 (LOAEL)	3,000	6.7	1.0	200
	[26]	Lowest daily therapeutic dose (10 mg/d).	Therapeutic effect.	166.7 (LDTD)	1,000	0.17	1.0	5.0
Azithromycin (antibiotic)	[12]	Lowest daily therapeutic dose (500 mg/d).	Therapeutic effect.	8,330 (LDTD)	1,000	8.3	0.1	25
	[26,27]	Estimation of the minimal inhibitory concentration (MIC50 = 3.74 µg/mL) of intestinal flora, according to EMEA methodology [28].	Intestinal flora alterations in humans (tulathromycin).	-	-	3	0.1	9.0
Amlodipine besilate (antihypertensive)	[24]	Lowest daily therapeutic dose (5 mg/d).	Therapeutic effect.	83.3 (LDTD)	1,000	0.08	1.0	2.5
Betamethasone (steroidal anti-inflammatory)	[24]	Lowest daily therapeutic dose (0.5 mg/d).	Therapeutic effect.	8.3 (LDTD)	1,000	0.008	1.0	0.25

ADI: Acceptable daily intake; AF: allocation factor of the ADI for the exposure via drinking water consumption; UF: uncertainty factor; NOAEL: no-observed-adverse-effect levels: highest level of exposure for which no adverse effects are observed; LOAEL: lowest-observed-adverse-effect levels: lowest exposure level for which adverse effects are observed; LDTD: Lowest daily therapeutic dose of a drug; BMDL10: lower limit of critical effect of the benchmark dose (related to the lower limit at a 95% confidence interval for the level of exposure that increases by 10% the risk below the least adverse effect).

[1] The values do not correspond to the original references since in this work they were calculated considering specific premises and factors adjusted to the Brazilian reality (see item 2.2 - Estimation of guideline values for P&EDC in drinking waters).

Table S4 cont. Toxicological studies and guideline values (GV) for selected pharmaceutical and endocrine disrupting compounds (P&EDC).

CEC	Reference	Type of study	Critical effect	Experimental dose (µg/kg bw.d)	UF	ADI (µg/kg bw.d)	AF[1]	GV[1] (µg/L)
Bezafibrate (antilipemic)	[26,29]	Lowest daily therapeutic dose (600 mg/d).	Therapeutic effect.	10,000 (LDTD)	1,000	10	1.0	300
Bisphenol A (chemical input)	[26,30]	Chronic bioassay in rats.	Decreases in food consumption and body weight.	50,000 (LOAEL)	1,000	50	0.6	900
	[12]	Three-generational study in rats.	Decreases in body weight.	5,000 (NOAEL)	100	50	0.6	900
	[31]	Toxicokinetic study based on the lowest limit of the benchmark dose - BMDL10 which cause effects in rats.	Effects on kidney.	609 ($BMDL_{10}$ human equivalent dose)	150	4	0.6	72
Cefalexin - CEF (antibiotic)	[26]	Estimation of the minimal inhibitory concentration of intestinal flora, according to EMEA methodology [28].	Intestinal flora alterations in humans.	-	-	10	0.1	30
Cimetidine (antiulcer)	[26,32]	Lowest daily therapeutic dose (200 mg/d).	Therapeutic effect.	3,300 (LDTD)	1,000	3.3	1.0	100

ADI: Acceptable daily intake; AF: allocation factor of the ADI for the exposure via drinking water consumption; UF: uncertainty factor; NOAEL: no-observed-adverse-effect levels: highest level of exposure for which no adverse effects are observed; LOAEL: lowest-observed-adverse-effect levels: lowest exposure level for which adverse effects are observed; LDTD: Lowest daily therapeutic dose of a drug; BMDL10: lower limit of critical effect of the benchmark dose (related to the lower limit at a 95% confidence interval for the level of exposure that increases by 10% the risk below the least adverse effect).

[1] The values do not correspond to the original references since in this work they were calculated considering specific premises and factors adjusted to the Brazilian reality (see item 2.2 - Estimation of guideline values for P&EDC in drinking waters).

Table S4 cont. Toxicological studies and guideline values (GV) for selected pharmaceutical and endocrine disrupting compounds (P&EDC).

CEC	Reference	Type of study	Critical effect	Experimental dose (µg/kg bw.d)	UF	ADI (µg/kg bw.d)	AF[1]	GV[1] (µg/L)
Ciprofloxacin (antibiotic)	[26]	Lowest daily therapeutic dose (500 mg/d).	Therapeutic effect.	8,330 (LDTD)	1,000	8.3	0.1	25
	[32]	Estimation of the minimal inhibitory concentration (MIC50 = 0.0016 µg/mL) of intestinal flora, according to EMEA methodology [28].	Intestinal flora alterations in humans.	-	-	1.6	0.1	4.8
Clarithromycin (antibiotic)	[24]	Lowest daily therapeutic dose (500 mg/d).	Therapeutic effect.	8,330 (LDTD)	1,000	8.3	1.0	250
Clonazepam (anxyolitic)	[24]	Lowest daily therapeutic dose (0.5 mg/d).	Therapeutic effect.	8.3	1,000	0.008	1.0	0.25
Dexamethasone (steroidal anti-inflammatory)	[33]	Subchronic study in rats.	Induction of tyrosine aminotransferase activity in liver.	1.5	100	0.015	0.1	0.045
	[24]	Lowest daily therapeutic dose (0.5 mg/d).	Therapeutic effect.	8.3 (LDTD)	1,000	0.008	0.1	0.025

ADI: Acceptable daily intake; AF: allocation factor of the ADI for the exposure via drinking water consumption; UF: uncertainty factor; NOAEL: no-observed-adverse-effect levels: highest level of exposure for which no adverse effects are observed; LOAEL: lowest-observed-adverse-effect levels: lowest exposure level for which adverse effects are observed; LDTD: Lowest daily therapeutic dose of a drug; BMDL10: lower limit of critical effect of the benchmark dose (related to the lower limit at a 95% confidence interval for the level of exposure that increases by 10% the risk below the least adverse effect).

[1] The values do not correspond to the original references since in this work they were calculated considering specific premises and factors adjusted to the Brazilian reality (see item 2.2 - Estimation of guideline values for P&EDC in drinking waters).

Table S4 cont. Toxicological studies and guideline values (GV) for selected pharmaceutical and endocrine disrupting compounds (P&EDC).

CEC	Reference	Type of study	Critical effect	Experimental dose (µg/kg bw.d)	UF	ADI (µg/kg bw.d)	AF[1]	GV[1] (µg/L)
Diclofenac (nonsteroidal anti-inflammatory)	[12]	Toxicological study in rats which assessed the gestation period.	Developmental.	20,000 (NOAEL)	300	67	0.1	200
	[12]	Lowest daily therapeutic dose (100 mg/d).	Therapeutic effect.	1,700 (LDTD)	1,000	1.7	0.1	5.0
	[26]	Reproductive toxicity studies in rats.	Effects on fetal development.	0.1 (LOAEL)	200	0.50	0.1	1.5
Diltiazem (antihypertensive)	[26]	Lowest daily therapeutic dose (120 mg/d).	Therapeutic effect.	2,000 (LDTD)	1,000	2	1.0	60
Dipyrone (analgesic)	[26,34]	Studies in dogs.	Behavioral and hematological alterations and decreases in body weight gain.	30,000 (NOAEL)	200	150	0.1	450
	[24]	Lowest daily therapeutic dose (500 mg/d).	Therapeutic effect.	8,330 (LDTD)	1,000	8.3	0.1	25
Enalapril (antihypertensive)	[12]	Epidemiological study which assessed the gestation period.	Developmental.	70 (LOAEL)	300	0.23	1.0	7
	[24]	Lowest daily therapeutic dose (2.5 mg/d).	Therapeutic effect.	42 (LDTD)	1,000	0.04	1.0	1.3

ADI: Acceptable daily intake; AF: allocation factor of the ADI for the exposure via drinking water consumption; UF: uncertainty factor; NOAEL: no-observed-adverse-effect levels: highest level of exposure for which no adverse effects are observed; LOAEL: lowest-observed-adverse-effect levels: lowest exposure level for which adverse effects are observed; LDTD: Lowest daily therapeutic dose of a drug; BMDL10: lower limit of critical effect of the benchmark dose (related to the lower limit at a 95% confidence interval for the level of exposure that increases by 10% the risk below the least adverse effect).

[1] The values do not correspond to the original references since in this work they were calculated considering specific premises and factors adjusted to the Brazilian reality (see item 2.2 - Estimation of guideline values for P&EDC in drinking waters).

Table S4 cont. Toxicological studies and guideline values (GV) for selected pharmaceutical and endocrine disrupting compounds (P&EDC).

CEC	Reference	Type of study	Critical effect	Experimental dose (µg/kg bw.d)	UF	ADI (µg/kg bw.d)	AF[1]	GV[1] (µg/L)
Enoxacin (antibiotic)	[24]	Lowest daily therapeutic dose (400 mg/d).	Therapeutic effect.	6,667 (LDTD)	1,000	6.7	1.0	200
Enrofloxacin (antibiotic)	[26]	Not reported.	Not reported.	Not reported.	Not reported.	6.2	0.1	20
17-beta-Estradiol (estrogen)	[12,26,35]	Epidemiological study of post-menopausal women receiving hormonal therapy.	Serum concentrations of follicle-stimulating hormone, angiotensinogen, sex hormone-binding globulin, and corticosteroid-binding globulin.	5 (NOAEL)	100	0.050	0.2	0.30
	[36]	Dose-response studies in rats.	Development of breast, uterus, and mesentery cancers.	1/39,000 (slope factor)	10^{-5} (cancer risk)	-	-	0.008
Estrone (estrogen)	[36]	Epidemiological study of post-menopausal women receiving hormonal therapy.	Estrogenic response.	5 (NOAEL)	100	0.050	0.2	0.30
	[12]	2-Week study in humans.	Effects on endocrine system and liver.	4 (NOAEL)	300	0.013	0.2	0.078
	[26]	Lowest daily therapeutic dose (0.6 mg/d).	Tulathromycin.	10 (LDTD)	10,000	0.001	0.2	0.006

ADI: Acceptable daily intake; AF: allocation factor of the ADI for the exposure via drinking water consumption; UF: uncertainty factor; NOAEL: no-observed-adverse-effect levels: highest level of exposure for which no adverse effects are observed; LOAEL: lowest-observed-adverse-effect levels: lowest exposure level for which adverse effects are observed; LDTD: Lowest daily therapeutic dose of a drug; BMDL10: lower limit of critical effect of the benchmark dose (related to the lower limit at a 95% confidence interval for the level of exposure that increases by 10% the risk below the least adverse effect).[1] The values do not correspond to the original references since in this work they were calculated considering specific premises and factors adjusted to the Brazilian reality (see item 2.2 - Estimation of guideline values for P&EDC in drinking waters).

Table S4 cont. Toxicological studies and guideline values (GV) for selected pharmaceutical and endocrine disrupting compounds (P&EDC).

CEC	Reference	Type of study	Critical effect	Experimental dose (µg/kg bw.d)	UF	ADI (µg/kg bw.d)	AF[1]	GV[1] (µg/L)
Estriol (estrogen)	[36]	Epidemiological study of post-menopausal women receiving hormonal therapy.	Serum concentrations of follicle-stimulating hormone, angiotensinogen, sex hormone-binding globulin, and corticosteroid-binding globulin.	5 (NOAEL)	100	0.050	0.2	0.30
	[26]	Lowest daily therapeutic dose (1.0 mg/d).	Therapeutic effect.	16.7 (LDTD)	10,000	0.0017	0.2	0.010
17-alpha-Ethinylestradiol (estrogen)	[36]	Study in humans.	Increased serum levels of alanine aminotransferase, aspartate aminotransferase, and gamma glutamyl transferase.	15 (LOAEL)	3,000	0.0050	1.0	0.15
	[12]	Study in humans.	Effects on endocrine system.	0.1 (NOAEL)	1,000	0.00010	1.0	0.0030
	[26]	Lowest daily therapeutic dose (0.03 mg/d).	Therapeutic effect.	0.5 (LDTD)	10,000	0.00005	1.0	0.0015
Fluconazole (antifungal)	[24]	Lowest daily therapeutic dose (50 mg/d).	Therapeutic effect.	830 (LDTD)	1,000	0.8	1.0	25

ADI: Acceptable daily intake; AF: allocation factor of the ADI for the exposure via drinking water consumption; UF: uncertainty factor; NOAEL: no-observed-adverse-effect levels: highest level of exposure for which no adverse effects are observed; LOAEL: lowest-observed-adverse-effect levels: lowest exposure level for which adverse effects are observed; LDTD: Lowest daily therapeutic dose of a drug; BMDL10: lower limit of critical effect of the benchmark dose (related to the lower limit at a 95% confidence interval for the level of exposure that increases by 10% the risk below the least adverse effect).

[1] The values do not correspond to the original references since in this work they were calculated considering specific premises and factors adjusted to the Brazilian reality (see item 2.2 - Estimation of guideline values for P&EDC in drinking waters).

Table S4 cont. Toxicological studies and guideline values (GV) for selected pharmaceutical and endocrine disrupting compounds (P&EDC).

CEC	Reference	Type of study	Critical effect	Experimental dose (µg/kg bw.d)	UF	ADI (µg/kg bw.d)	AF[1]	GV[1] (µg/L)
Gemfibrozil (antilipemic)	[12]	Toxicological study in rats which assessed the gestation and postnatal period.	Developmental.	92,000 (LOAEL)	3,000	31	1.0	930
	[26]	Lowest daily therapeutic dose (1,200 mg/d).	Therapeutic effect.	20,000 (LDTD)	1,000	20	1.0	600
Hydrochlorothiazide (antihypertensive)	[24]	Lowest daily therapeutic dose (12.5 mg/d).	Therapeutic effect.	208 (LDTD)	1,000	0.21	0.1	0.6
Ibuprofen (nonsteroidal anti-inflammatory)	[26]	Lowest daily therapeutic dose (800 mg/d).	Therapeutic effect.	13,300 (LDTD)	1,000	13.3	1.0	400
Ketoprofen (nonsteroidal anti-inflammatory)	[26]	Not reported.	Not reported.	-	-	1.0	0.1	3.0
	[24]	Lowest daily therapeutic dose (100 mg/d).	Therapeutic effect.	1,700 (LDTD)	1,000	1.7	0.1	5.0
Levonorgestrel (hormonal contraceptive)	[24]	Lowest daily therapeutic dose (0.03 mg/d).	Therapeutic effect.	0.5 (LDTD)	1,000	0.0005	1.0	0.015

ADI: Acceptable daily intake; AF: allocation factor of the ADI for the exposure via drinking water consumption; UF: uncertainty factor; NOAEL: no-observed-adverse-effect levels: highest level of exposure for which no adverse effects are observed; LOAEL: lowest-observed-adverse-effect levels: lowest exposure level for which adverse effects are observed; LDTD: Lowest daily therapeutic dose of a drug; BMDL10: lower limit of critical effect of the benchmark dose (related to the lower limit at a 95% confidence interval for the level of exposure that increases by 10% the risk below the least adverse effect).

[1] The values do not correspond to the original references since in this work they were calculated considering specific premises and factors adjusted to the Brazilian reality (see item 2.2 - Estimation of guideline values for P&EDC in drinking waters).

Table S4 cont. Toxicological studies and guideline values (GV) for selected pharmaceutical and endocrine disrupting compounds (P&EDC).

CEC	Reference	Type of study	Critical effect	Experimental dose (µg/kg bw.d)	UF	ADI (µg/kg bw.d)	AF[1]	GV[1] (µg/L)
Levothyroxine (Synthetic hormone)	[24]	Lowest daily therapeutic dose (0.0125 mg/d).	Therapeutic effect.	0.21 (LDTD)	1,000	0.0002	1.0	0.006
Linezolid (antibiotic)	[24]	Lowest daily therapeutic dose (800 mg/d).	Therapeutic effect.	13,300 (LDTD)	1,000	13.3	1.0	400
Loratadine (antiallergic)	[24]	Lowest daily therapeutic dose (10 mg/d).	Therapeutic effect.	167 (LDTD)	1,000	0.17	1.0	5.0
Losartan (antihypertensive)	[24]	Lowest daily therapeutic dose (25 mg/d).	Therapeutic effect.	415 (LDTD)	1,000	0.42	1.0	13
Metformin (antidiabetic)	[26]	Lowest daily therapeutic dose (500 mg/d).	Therapeutic effect.	8,330 (LDTD)	1,000	8.3	1.0	250
Naproxen (nonsteroidal anti-inflammatory)	[26]	Lowest daily therapeutic dose (440 mg/d).	Therapeutic effect.	7,300 (LDTD)	1,000	7.3	1.0	220
Nimesulide (anti-inflammatory)	[24]	Lowest daily therapeutic dose (200 mg/d).	Therapeutic effect.	3,330 (LDTD)	1,000	3.3	1.0	100

ADI: Acceptable daily intake; AF: allocation factor of the ADI for the exposure via drinking water consumption; UF: uncertainty factor; NOAEL: no-observed-adverse-effect levels: highest level of exposure for which no adverse effects are observed; LOAEL: lowest-observed-adverse-effect levels: lowest exposure level for which adverse effects are observed; LDTD: Lowest daily therapeutic dose of a drug; BMDL10: lower limit of critical effect of the benchmark dose (related to the lower limit at a 95% confidence interval for the level of exposure that increases by 10% the risk below the least adverse effect).

[1] The values do not correspond to the original references since in this work they were calculated considering specific premises and factors adjusted to the Brazilian reality (see item 2.2 - Estimation of guideline values for P&EDC in drinking waters).

Table S4 cont. Toxicological studies and guideline values (GV) for selected pharmaceutical and endocrine disrupting compounds (P&EDC).

CEC	Reference	Type of study	Critical effect	Experimental dose (µg/kg bw.d)	UF	ADI (µg/kg bw.d)	AF[1]	GV[1] (µg/L)
Nonylphenol (chemical input)	[12]	Three-generational study in rats.	Decreases in body and organ weights.	1,500 (NOAEL)	30	50	0.2	300
	[26,36,37]	Multigenerational study in rats.	Effects on reproductive system, including on the offspring.	15,000 (NOAEL)	1,000	15	0.2	90
Norfloxacin (antibiotic)	[26]	Lowest daily therapeutic dose (800 mg/d).	Therapeutic effect.	13,300 (LDTD)	1,000	13.3	0.1	40
Octylphenol (chemical input)	[12,26]	Three-generational study in rats.	Behavioral.	15,000 (LOAEL)	1,000	15	0.2	90
Omeprazole (antiulcer)	[24]	Lowest daily therapeutic dose (10 mg/d).	Therapeutic effect.	166.7 (LDTD)	1,000	0.17	1.0	5.0
Paracetamol / Acetaminophen (analgesic / antipyretic)	[26]	Not reported.	Not reported.	50,000 (NOAEL)	1,000	50	1.0	1,500
	[38]	The lowest daily therapeutic dose (325 mg/d) was lower than the NOAEL from toxicological studies which assessed effects on kidney and liver.	Therapeutic effect.	5,400 (LDTD)	1,000	5.4	1.0	160

ADI: Acceptable daily intake; AF: allocation factor of the ADI for the exposure via drinking water consumption; UF: uncertainty factor; NOAEL: no-observed-adverse-effect levels: highest level of exposure for which no adverse effects are observed; LOAEL: lowest-observed-adverse-effect levels: lowest exposure level for which adverse effects are observed; LDTD: Lowest daily therapeutic dose of a drug; BMDL10: lower limit of critical effect of the benchmark dose (related to the lower limit at a 95% confidence interval for the level of exposure that increases by 10% the risk below the least adverse effect).

[1] The values do not correspond to the original references since in this work they were calculated considering specific premises and factors adjusted to the Brazilian reality (see item 2.2 - Estimation of guideline values for P&EDC in drinking waters).

Table S4 cont. Toxicological studies and guideline values (GV) for selected pharmaceutical and endocrine disrupting compounds (P&EDC).

CEC	Reference	Type of study	Critical effect	Experimental dose (µg/kg bw.d)	UF	ADI (µg/kg bw.d)	AF[1]	GV[1] (µg/L)
Prednisone (steroidal anti-inflammatory)	[24]	Lowest daily therapeutic dose (2.5 mg/d).	Therapeutic effect.	42 (LDTD)	1,000	0.042	0.1	0.13
Promethazine (antihistamine)	[24]	Lowest daily therapeutic dose (20 mg/d).	Therapeutic effect.	330 (LDTD)	1,000	0.33	1.0	10
Propanolol (antihypertensive)	[24]	Lowest daily therapeutic dose (30 mg/d).	Therapeutic effect.	500 (LDTD)	1,000	0.5	1.0	15
Ranitidine (antiulcer)	[32]	Lowest daily therapeutic dose (75 mg/d).	Therapeutic effect.	1,250 (LDTD)	1,000	1.3	1.0	38
Sildenafil (phosphodiester-ase inhibitor)	[24]	Lowest daily therapeutic dose (12.5 mg/d).	Therapeutic effect.	208 (LDTD)	1,000	0.21	1.0	6.3
Simvastatin (antilipemic)	[12]	Epidemiological study of children.	Developmental.	200 (LOAEL)	300	0.7	1.0	20
	[24]	Lowest daily therapeutic dose (5 mg/d).	Therapeutic effect.	83.3 (LDTD)	1,000	0.08	1.0	2.5

ADI: Acceptable daily intake; AF: allocation factor of the ADI for the exposure via drinking water consumption; UF: uncertainty factor; NOAEL: no-observed-adverse-effect levels: highest level of exposure for which no adverse effects are observed; LOAEL: lowest-observed-adverse-effect levels: lowest exposure level for which adverse effects are observed; LDTD: Lowest daily therapeutic dose of a drug; BMDL10: lower limit of critical effect of the benchmark dose (related to the lower limit at a 95% confidence interval for the level of exposure that increases by 10% the risk below the least adverse effect).

[1] The values do not correspond to the original references since in this work they were calculated considering specific premises and factors adjusted to the Brazilian reality (see item 2.2 - Estimation of guideline values for P&EDC in drinking waters).

Table S4 cont. Toxicological studies and guideline values (GV) for selected pharmaceutical and endocrine disrupting compounds (P&EDC).

CEC	Reference	Type of study	Critical effect	Experimental dose (µg/kg bw.d)	UF	ADI (µg/kg bw.d)	AF[1]	GV[1] (µg/L)
Sulfamethoxazole (antibiotic)	[12]	Toxicological study in rats which assessed the gestation period.	Developmental.	512,000 (NOAEL)	1,000	512	0.1	1,535
	[25,32]	60-Week study in rats.	Tumors in thyroid.	25,000 (NOAEL)	200	130	0.1	390
	[12]	Lowest daily therapeutic dose (800 mg/d).	Therapeutic effect.	13,300 (LDTD)	1,000	13.3	0.1	40
	[26,39]	90-Day study in dogs.	Effects on thyroid.	1,000 (NOAEL)	100	10	0.1	30
Tetracycline (antibiotic)	[40]	Study in humans which assessed effects in intestinal flora and induction of resistant strains.	Selection of resistant Enterobacteriaceae strains.	33 (NOAEL)	1	30	0.1	90
Triclosan (antiseptic)	[41]	2-Year chronic study in hamsters.	Systemic effects.	75,000 (NOAEL)	1,000	75	0.1	225
	[38]	13-Week subchronic study in rats.	Effects on kidney and liver.	50,000 (NOAEL)	1,000	50	0.1	150

ADI: Acceptable daily intake; AF: allocation factor of the ADI for the exposure via drinking water consumption; UF: uncertainty factor; NOAEL: no-observed-adverse-effect levels: highest level of exposure for which no adverse effects are observed; LOAEL: lowest-observed-adverse-effect levels: lowest exposure level for which adverse effects are observed; LDTD: Lowest daily therapeutic dose of a drug; BMDL10: lower limit of critical effect of the benchmark dose (related to the lower limit at a 95% confidence interval for the level of exposure that increases by 10% the risk below the least adverse effect).

[1] The values do not correspond to the original references since in this work they were calculated considering specific premises and factors adjusted to the Brazilian reality (see item 2.2 - Estimation of guideline values for P&EDC in drinking waters).

Table S4 cont. Toxicological studies and guideline values (GV) for selected pharmaceutical and endocrine disrupting compounds (P&EDC).

CEC	Reference	Type of study	Critical effect	Experimental dose (µg/kg bw.d)	UF	ADI (µg/kg bw.d)	AF[1]	GV[1] (µg/L)
Trimethoprim (antibiotic)	[12]	Toxicological study in rats which assessed the gestation period.	Developmental.	192,000 (NOAEL)	1,000	192	0.1	575
	[26]	Not reported.	Not reported.	Not reported.	Not reported.	20	0.1	60
	[32]	Estimation of the minimal inhibitory concentration of intestinal flora, according to EMEA methodology [28].	Intestinal flora alterations in humans.	-	-	4.2	0.1	13
	[42]	Lowest daily therapeutic dose (100 mg/d).	Therapeutic effect.	1,670 (LDTD)	1,000	1.7	0.1	5.0

ADI: Acceptable daily intake; AF: allocation factor of the ADI for the exposure via drinking water consumption; UF: uncertainty factor; NOAEL: no-observed-adverse-effect levels: highest level of exposure for which no adverse effects are observed; LOAEL: lowest-observed-adverse-effect levels: lowest exposure level for which adverse effects are observed; LDTD: Lowest daily therapeutic dose of a drug; BMDL10: lower limit of critical effect of the benchmark dose (related to the lower limit at a 95% confidence interval for the level of exposure that increases by 10% the risk below the least adverse effect).

[1] The values do not correspond to the original references since in this work they were calculated considering specific premises and factors adjusted to the Brazilian reality (see item 2.2 - Estimation of guideline values for P&EDC in drinking waters).

Table S5. Margin of exposure to contaminants of emerging concern (CEC) in Brazilian waters.

Ranking	Compound	Class	ME[1]	Risk classification[2]
1º	17-alpha-Ethinylestradiol	Synthetic hormone	0.005	High
2º	Dexamethasone	Anti-inflammatory	0.01	High
3º	Prednisone	Anti-inflammatory	0.02	High
4º	Betamethasone	Anti-inflammatory	0.10	High
5º	17-beta-Estradiol	Natural hormone	0.18	High
6º	Naproxen	Anti-inflammatory	0.59	High
7º	Estrone	Natural hormone	0.82	High
8º	Diclofenac	Anti-inflammatory	1.1	Alert
9º	Trimethoprim	Antibiotic	1.1	Alert
10º	Propanolol	Antihypertensive	2.2	Alert
11º	Estriol	Natural hormone	3.1	Alert
12º	Acetylsalicylic acid	Analgesic	4.2	Alert
13º	Ketoprofen	Anti-inflammatory	5.3	Alert
14º	Atorvastatin	Antilipemic	7.6	Alert
15º	Sulfamethoxazole	Antibiotic	11.6	Moderate
16º	Levonorgestrel	Hormonal contraceptive	15.0	Moderate
17º	Losartan	Antihypertensive	22.6	Moderate
18º	Bisphenol A	Plasticizer	28.2	Moderate
19º	4-Nonylphenol	Chemical input	31.9	Moderate
20º	Fluconazole	Antifulgal	33.3	Moderate
21º	Amoxicilin	Antibiotic	47.6	Moderate
22º	Loratadine	Antihistamine	74.6	Moderate
23º	Enrofloxacin	Antibiotic	91.3	Moderate
24º	Bezafibrate	Antilipemic	180.8	Low
25º	Norfloxacin	Antibiotic	190.5	Low
26º	Gemfibrozil	Antilipemic	266.3	Low
27º	Omeprazole	Antiulcer	280.9	Low
28º	Promethazine	Antihypertensive	324.3	Low
29º	Paracetamol/Acetaminophen	Analgesic	352.7	Low
30º	4-Octylphenol	Chemical input	383.0	Low
31º	Linezolid	Antibiotic	443.9	Low
32º	Enoxacin	Antibiotic	498.0	Low
33º	Ibuprofen	Anti-inflammatory	816.0	Low
34º	Ranitidine	Antiulcer	1,423.2	Very low
35º	Atenolol	Antihypertensive	1,724.1	Very low
36º	Metformin	Antidiabetic	2,248.2	Very low
37º	Cimetidine	Antiulcer	3,378.4	Very low
38º	Acyclovir	Antiviral	4,297.4	Very low
39º	Clarithromycin	Antibiotic	7,692.3	Very low
40º	Diltiazem	Antihypertensive	49,180.3	Very low
41º	Triclosan	Antiseptic	50,000.0	Very low

[1] ME = guideline value (lowest reported) ÷ occurrence (maximum concentration in drinking water). [2] ME<1: high risk; ME between 1 and 10: alert; ME between 10 and 100: moderate risk; ME between 100 and 1.000 :low risk; ME >1.000 negligible risk.

References

1. ANVISA. 2017 Pharmaceutical Market Statistical Yearbook (in Portuguese). Available online: http://antigo.anvisa.gov.br/documents/374947/3413536/Anu%C3%A1rio+Estat%C3%ADstico+do+Mercado+Farmac%C3%AAutico+-+2018/c24aacbf-4d0c-46a7-bb86-b92c170c83e1 (accessed on 29 May 2021).
2. Lima, D.R.S.; Tonucci, M.C.; Libânio, M.; Aquino, S.F. Pharmaceuticals and endocrine disrupting compounds in Brazilian waters: occurrence and removal techniques (in Portuguese). *Eng. Sanit. Ambient.* **2017**, *22*, 1043-1054.
3. Reis, E.O.; Foureaux, A.F.S.; Rodrigues, J.S.; Moreira, V.R.; Lebron, Y.A.R.; Santos, L.V.S.; Amaral, M.C.S.; Lange, L.C. Occurrence, removal and seasonal variation of pharmaceuticals in Brasilian drinking water treatment plants. *Environ. Pollut.* **2019**, *250*, 773-781.
4. Brandt, E.M.F.; Aquino, S.F.; Bastos, R.K.X. *Review of the Annex XX of Consolidation Ordinance No. 5 of September 28, 2017 of the Ministry of Health (former Ordinance MS No. 2914/2011). Theme II - Drinking water standard and sampling plans. Chemical substances – Pharmaceuticals and endocrine disruptors. Subsidies for discussion and review guidelines (in Portuguese)*; Ministério da Saúde: Brasília, DF, 2019.
5. Zini, L.B.; Gutterres, M. Chemical contaminants in Brazilian drinking water: a systematic review. *J. Water Health* **2021**, *19*, 351-369.
6. Machado, K.C.; Grassi, M.T.; Vidal, C.; Pescara, I.C.; Jardim, W.F.; Fernandes, A.N.; Sodré, F.F.; Almeida, F.V.; Santana, J.S.; Canela, M.C.; et al. A preliminary nationwide survey of the presence of emerging contaminants in drinking and source waters in Brazil. *Sci. Total Environ.* **2016**, *572*, 138-146.
7. Llamas-Dios, M.I.; Vadillo, I.; Jiménez-Gavilán, P.; Candela, L.; Corada-Fernández, C. Assessment of a wide array of contaminants of emerging concern in a Mediterranean water basin (Guadalhorce river, Spain): Motivations for an improvement of water management and pollutants surveillance. *Sci. Total Environ.* **2021**, *788*, 147822.
8. Baken, K.A.; Sjerps, R.M.A.; Schriks, M.; van Wezel, A.P. Toxicological risk assessment and prioritization of drinking water relevant contaminants of emerging concern. *Environ. Int.* **2018**, *118*, 293-303.
9. Patel, M.; Kumar, R.; Kishor, K.; Mlsna, T.; Pittman, C.U., Jr.; Mohan, D. Pharmaceuticals of Emerging Concern in Aquatic Systems: Chemistry, Occurrence, Effects, and Removal Methods. *Chem. Rev.* **2019**, *119*, 3510-3673.
10. Funke, J.; Prasse, C.; Ternes, T.A. Identification of transformation products of antiviral drugs formed during biological wastewater treatment and their occurrence in the urban water cycle. *Water Res.* **2016**, *98*, 75-83.
11. Riva, F.; Castiglioni, S.; Fattore, E.; Manenti, A.; Davoli, E.; Zuccato, E. Monitoring emerging contaminants in the drinking water of Milan and assessment of the human risk. *Int. J. Hyg. Environ. Health* **2018**, *221*, 451-457.
12. Awwa Research Foundation. *Removal of EDCs and pharmaceuticals in drinking and reuse treatment processes*; Snyder, S.A., Wert, E.C., Lei, H.D., Westerhoff, P., Yoon, Y., Eds.; AwwaRF: Denver, Colorado, 2007.
13. Huerta-Fontela, M.; Galceran, M.T.; Ventura, F. Occurrence and removal of pharmaceuticals and hormones through drinking water treatment. *Water Res.* **2011**, *45*, 1432-1442.
14. Valbonesi, P.; Profita, M.; Vasumini, I.; Fabbri, E. Contaminants of emerging concern in drinking water: Quality assessment by combining chemical and biological analysis. *Sci. Total Environ.* **2021**, *758*, 143624.
15. Tröger, R.; Ren, H.; Yin, D.; Postigo, C.; Nguyen, P.D.; Baduel, C.; Golovko, O.; Been, F.; Joerss, H.; Boleda, M.R.; et al. What's in the water? – Target and suspect screening of contaminants of emerging concern in raw water and drinking water from Europe and Asia. *Water Res.* **2021**, *198*, 117099.
16. Angeles, L.F.; Islam, S.; Aldstadt, J.; Saqeeb, K.N.; Alam, M.; Khan, M.A.; Johura, F.-T.; Ahmed, S.I.; Aga, D.S. Retrospective suspect screening reveals previously ignored antibiotics, antifungal compounds, and metabolites in Bangladesh surface waters. *Sci. Total Environ.* **2020**, *712*, 136285.
17. Batt, A.L.; Kostich, M.S.; Lazorchak, J.M. Analysis of Ecologically Relevant Pharmaceuticals in Wastewater and Surface Water

Using Selective Solid-Phase Extraction and UPLC−MS/MS. *Anal. Chem.* **2008**, *80*, 5021-5030.

18. Shen, X.; Chang, H.; Sun, Y.; Wan, Y. Determination and occurrence of natural and synthetic glucocorticoids in surface waters. *Environ. Int.* **2020**, *134*, 105278.
19. Weizel, A.; Schlüsener, M.P.; Dierkes, G.; Ternes, T.A. Occurrence of Glucocorticoids, Mineralocorticoids, and Progestogens in Various Treated Wastewater, Rivers, and Streams. *Environ. Sci. Technol.* **2018**, *52*, 5296-5307.
20. Stackelberg, P.E.; Furlong, E.T.; Meyer, M.T.; Zaugg, S.D.; Henderson, A.K.; Reissman, D.B. Persistence of pharmaceutical compounds and other organic wastewater contaminants in a conventional drinking-water-treatment plant. *Sci. Total Environ.* **2004**, *329*, 99-113.
21. Singh, R.R.; Angeles, L.F.; Butryn, D.M.; Metch, J.W.; Garner, E.; Vikesland, P.J.; Aga, D.S. Towards a harmonized method for the global reconnaissance of multi-class antimicrobials and other pharmaceuticals in wastewater and receiving surface waters. *Environ. Int.* **2019**, *124*, 361-369.
22. Appa, R.; Mhaisalkar, V.A.; Bafana, A.; Saravana Devi, S.; Krishnamurthi, K.; Chakrabarti, T.; Naoghare, P.K. Simultaneous quantitative monitoring of four indicator contaminants of emerging concern (CEC) in different water sources of Central India using SPE/LC-(ESI)MS-MS. *Environ. Monit. Assess.* **2018**, *190*, 489.
23. Balakrishna, K.; Rath, A.; Praveenkumarreddy, Y.; Guruge, K.S.; Subedi, B. A review of the occurrence of pharmaceuticals and personal care products in Indian water bodies. *Ecotoxicol. Environ. Saf.* **2017**, *137*, 113-120.
24. Sweetman, S.C. *Martindale: the complete drug reference*, 36 ed.; Pharmaceutical Press: London; Chicago, 2009; p. 3694.
25. Schriks, M.; Heringa, M.B.; van der Kooi, M.M.E.; de Voogt, P.; van Wezel, A.P. Toxicological relevance of emerging contaminants for drinking water quality. *Water Res.* **2010**, *44*, 461-476.
26. EPHC/NRMMC/AHMC. *Australian Guidelines for Water Recycling: Augmentation of Drinking Water Supplies*; EPHC/NRMMC/AHMC: Canberra, Australia, 2008.
27. EMEA. European Medicines Evaluation Agency. European public MRL assessment report (EPMAR) - Tulathromycin (modification of the microbiological ADI and MRLs in bovine and porcine species) – after provisional maximum residue limits (MRLs). Available online: https://www.ema.europa.eu/en/documents/mrl-report/tulathromycin-modification-microbiological-adi-mrls-bovine-porcine-species-after-provisional-maximum_en.pdf (accessed on 29 May 2021).
28. EMEA. European Medicines Evaluation Agency. *Enrofloxacin (modification for bovine, porcine and poultry). Summary report (2)*; Veterinary Medicines Evaluation Unit: London, 1998.
29. de Jongh, C.M.; Kooij, P.J.; de Voogt, P.; ter Laak, T.L. Screening and human health risk assessment of pharmaceuticals and their transformation products in Dutch surface waters and drinking water. *Sci. Total Environ.* **2012**, *427-428*, 70-77.
30. USEPA. United States Environmental Protection Agency. Bisphenol A; CASRN 80-05-7. Available online: https://cfpub.epa.gov/ncea/iris/iris_documents/documents/subst/0356_summary.pdf (accessed in 29 May 2021).
31. European Union. *Scientific opinion on bisphenol A*; EFSA – European Food Safety Authority: Parma, Italy, 2015.
32. Schwab, B.W.; Hayes, E.P.; Fiori, J.M.; Mastrocco, F.J.; Roden, N.M.; Cragin, D.; Meyerhoff, R.D.; D'Aco, V.J.; Anderson, P.D. Human pharmaceuticals in US surface waters: a human health risk assessment. *Regul. Toxicol. Pharmacol.* **2005**, *42*, 296-312.
33. FAO/WHO. Evaluations of the Joint FAO/WHO Expert Committee on Food Additives (JECFA) – Dexamethasone. Available online: http://www.inchem.org/documents/jecfa/jecmono/v33je09.htm (accessed on 29 May 2021).
34. EMEA. European Medicines Evaluation Agency. Metamizole – Summary report. Available online: https://www.ema.europa.eu/en/documents/mrl-report/metamizole-summary-report-2-committee-veterinary-medicinal-products_en.pdf (accessed on 29 May 2021).
35. FAO/WHO. Estradiol-17β, Progesterone, and Testosterone. Joint FAO/WHO Expert Committee on Food Additives (JECFA). Available online: http://www.inchem.org/documents/jecfa/jecmono/v43jec05.htm (accessed in 29 May 2021).
36. USEPA. United States Environmental Protection Agency. Contaminant Information Sheets for the Final CCL 4 Chemicals.

Available online: https://www.epa.gov/ccl/contaminant-candidate-list-4-ccl-4 (accessed on 29 May 2021).
37. European Union. *Opinion of the Scientific Committee on Food on Bisphenol A. SCF/CS/PM/3936 Final.*; Scientific Committee on Food: Brussel, Belgium, 2002.
38. Blanset, D.L.; Zhang, J.; Robson, M.G. Probabilistic Estimates of Lifetime Daily Doses from Consumption of Drinking Water Containing Trace Levels of N,N-diethyl-meta-toluamide (DEET), Triclosan, or Acetaminophen and the Associated Risk to Human Health. *Hum. Ecol. Risk Assess.* **2007**, *13*, 615-631.
39. NRA. National Registration Authority for Agricultural and Veterinary Chemicals. *The NRA Review of sulphonamides. Final Report. August 2000 NRA Review Series 00.3*; National Registration Authority for Agricultural Chemicals: Canberra, Australia, 2000.
40. FAO/WHO. Tetracyclines: Oxytetracycline, Chlortetracycline and Tetracycline (addendum) Joint FAO/WHO Expert Committee on Food Additives. Available online: http://www.inchem.org/documents/jecfa/jecmono/v041je07.htm (accessed on 29 May 2021).
41. Barbolt, T.A. Chemistry and safety of triclosan, and its use as an antimicrobial coating on Coated VICRYL* Plus Antibacterial Suture (coated polyglactin 910 suture with triclosan). *Surg. Infect. (Larchmt.)* **2002**, *3 Suppl 1*, S45-53.
42. Cunningham, V.L.; Binks, S.P.; Olson, M.J. Human health risk assessment from the presence of human pharmaceuticals in the aquatic environment. *Regul. Toxicol. Pharm.* **2009**, *53*, 39-45.
